# An integrated enhancement and reconstruction strategy for the quantitative extraction of actin stress fibers from fluorescence micrographs

**DOI:** 10.1186/s12859-017-1684-y

**Published:** 2017-05-22

**Authors:** Zhen Zhang, Shumin Xia, Pakorn Kanchanawong

**Affiliations:** 1 Mechanobiology Institute, Singapore, 117411 Republic of Singapore; 2 Department of Biomedical Engineering, National University of Singapore, Singapore, 117411 Republic of Singapore

**Keywords:** Stress Fiber, Actin cytoskeleton, TIRF, Segmentation, Filament tracing, Micropattern

## Abstract

**Background:**

The stress fibers are prominent organization of actin filaments that perform important functions in cellular processes such as migration, polarization, and traction force generation, and whose collective organization reflects the physiological and mechanical activities of the cells. Easily visualized by fluorescence microscopy, the stress fibers are widely used as qualitative descriptors of cell phenotypes. However, due to the complexity of the stress fibers and the presence of other actin-containing cellular features, images of stress fibers are relatively challenging to quantitatively analyze using previously developed approaches, requiring significant user intervention. This poses a challenge for the automation of their detection, segmentation, and quantitative analysis.

**Result:**

Here we describe an open-source software package, SFEX (Stress Fiber Extractor), which is geared for efficient enhancement, segmentation, and analysis of actin stress fibers in adherent tissue culture cells. Our method made use of a carefully chosen image filtering technique to enhance filamentous structures, effectively facilitating the detection and segmentation of stress fibers by binary thresholding. We subdivided the skeletons of stress fiber traces into piecewise-linear fragments, and used a set of geometric criteria to reconstruct the stress fiber networks by pairing appropriate fiber fragments. Our strategy enables the trajectory of a majority of stress fibers within the cells to be comprehensively extracted. We also present a method for quantifying the dimensions of the stress fibers using an image gradient-based approach. We determine the optimal parameter space using sensitivity analysis, and demonstrate the utility of our approach by analyzing actin stress fibers in cells cultured on various micropattern substrates.

**Conclusion:**

We present an open-source graphically-interfaced computational tool for the extraction and quantification of stress fibers in adherent cells with minimal user input. This facilitates the automated extraction of actin stress fibers from fluorescence images. We highlight their potential uses by analyzing images of cells with shapes constrained by fibronectin micropatterns. The method we reported here could serve as the first step in the detection and characterization of the spatial properties of actin stress fibers to enable further detailed morphological analysis.

**Electronic supplementary material:**

The online version of this article (doi:10.1186/s12859-017-1684-y) contains supplementary material, which is available to authorized users.

## Background

The stress fibers are prominent assemblies of filamentous actin (F-actin) commonly observed in adherent tissue culture cells. Often considered to be the parallels of the contractile sarcomeric units of muscles [1], each stress fiber arises from higher-order organization of >10-30 F-actin filaments, numerous actin cross-linking proteins, and non-muscle myosin II molecular motors [1–8]. The stress fibers generate substantial mechanical forces that power cellular contraction against the extracellular matrix [9–12]. Meanwhile, the formation, stability, dynamics, and morphology of the stress fibers are highly regulated by mechanical and biochemical cues [13–22]. For instance, upregulation of contractility, actin polymerization, and matrix adhesion promote the formation and thickening of stress fibers, whereas cell relaxation, the inhibition of contractility, and actin cytoskeletal disruption lead to their disassembly and disintegration [15, 17, 18, 20].

A typical adherent cell contains an ensemble of stress fibers that span between adhesion sites or interconnect with one another across the cells, forming an integrated contractility apparatus that plays central roles in morphodynamic programmes such as migration, adhesion, and polarization [21, 23–26]. Stress fiber organization therefore underpins important cellular behaviors involved in both normal and pathological processes including developmental morphogenesis and cancer metastasis [27–30]. Since the stress fibers can be readily visualized both in living or fixed cells, by fluorescence microscopy using F-actin targeting fluorophores [31–33], the architecture of the stress fiber network has long been recognized as a key phenotypic reporter of cellular physiology [34]. Nevertheless, although such images may encode valuable information on cellular signaling and mechanobiological states, in many studies the analysis of the stress fiber network architecture were often restricted to qualitative descriptions, in significant part due to the limited availability of appropriate methods for quantitative extraction and analysis of salient features of the stress fiber networks.

Stress fibers typically are observed as networks of numerous elongated filaments. The recognition of filamentous image features has been extensively explored in fields such as geospatial informatics, neurosciences and astrophysics [35–37]. However, due to large variations in imaging methods, feature complexity, image resolution, and noise level, methods developed for a given type of curvilinear structures may not be directly applicable to others. For stress fibers, several approaches have previously been developed for their characterization [12, 38–44]. For example, order parameters analysis has been used to describe the aggregate image texture and orientation, without explicit treatment of each discrete stress fibers [38], thus avoiding the challenging task of detecting and segmenting individual filaments. Alternatively, a simulation-based approach can be used to study the stress fiber networks based on Finite Element analysis of idealized cellular architectures [12, 40–45]. Likewise, stress fiber networks can be treated as a micrograph-based linear superposition of filaments such that relevant coefficients can be solved by linear optimization [40]. However, major limitations of these approaches are their inability to extract empirical characteristics such as the dimension, density, and interactions between filaments, which are of key biologically relevance in the study of actin cytoskeletal organization.

We note that an approach that enables the extraction of individual stress fibers is potentially highly beneficial, particularly as this would permit direct correlation between experiments and theoretical models, a key step towards quantitative and predictive understanding of the underlying mechanisms [40]. However, existing methods for discrete filaments extraction have been parametrized for sparsely distributed filaments in cell periphery, cytoskeleton networks polymerized in vitro, or super-resolution microscopy images where improved resolution permits visual distinction of filaments [46–55]. To our knowledge, a computational method for the identification and complete extraction of the stress fibers in fluorescence micrographs of cells has not been available.

**Fig. 1:**
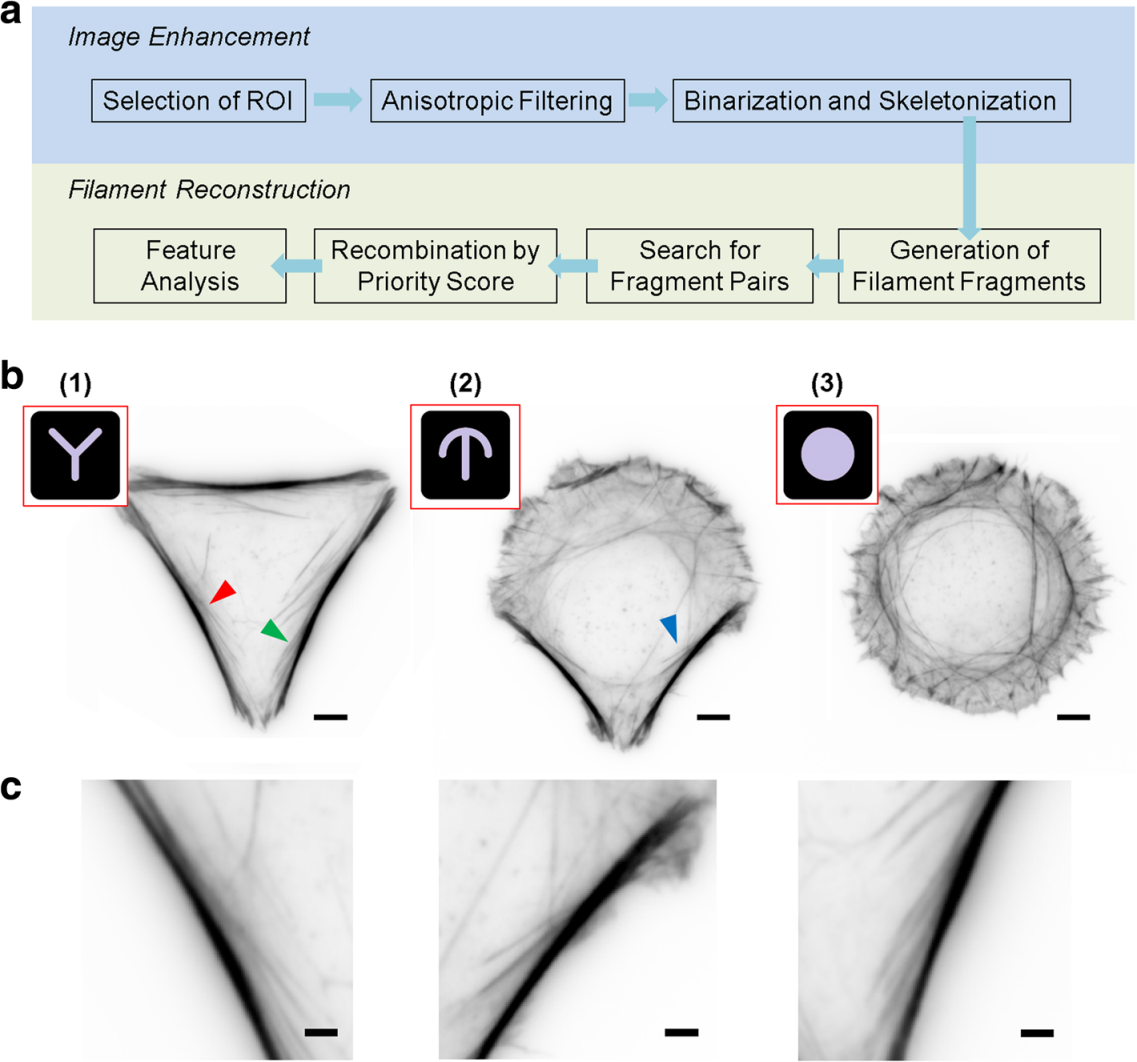
TIRFM images of F-actin and the analysis pipeline for stress fiber extraction **a** Analysis pipeline for image enhancement and segmentation of stress fibers. **b** TIRFM images of U2OS cells plated on Y- (1), crossbow- (2) and disc-shaped (3) micropatterns (red boxes) with red, green and blue arrows indicating regions of stress fiber branching. Scale bar, 5 μm. **c** Enlarged images of regions highlighted by red, green and blue arrows in (1) and (2) of (B). Scale bar, 2 μm

While the stress fibers are often the most prominent F-actin-containing cellular features, a number of technical factors pose significant challenges for their automated extraction. These include the complex organization of the filaments, such as filament intersection and convergence, and the presence of numerous F-actin-containing structures which may appear as bright puncta or as indistinct background intensity which reduces the local contrast of the stress fibers (Fig. 1b, c, Additional file 1: Figure S1). In this study we present a computational strategy to address these challenges, implemented as a package called SFEX (Stress Fibers Extractor) (see Additional files 2 and 3). In brief, this involves two major steps, linear structure enhancement and stress fiber reconstruction (Fig. 1a). In the first, neighborhood-based enhancement methods, line filter transform (LFT) and orientation filter transform (OFT) [56], were applied to the raw fluorescence image to selectively enhance the contrast of linear objects against different shape profiles, allowing detection and segmentation by binary thresholding. Subsequently, minimal linear filament fragments which represent the centerlines of detected stress fibers were generated from the skeletonized binary images. These were then recombined to reconstruct the traces of individual stress fibers. Carried out iteratively, this process permits extraction of the majority of stress fibers in cells, thus allowing the spatial attributes of both individual fibers and their collective architecture to be determined. Altogether, SFEX enables the automated extraction of the fiber networks from fluorescence micrographs, and thus may facilitate large-scale quantitative analysis of actin stress fiber profiles for dissecting molecular mechanisms or in high-throughput screening applications.

## Methods

### Cell Culture and specimen preparation

Human Osteosarcoma Cells (U2OS) were obtained from the American Type Culture Collection (ATCC, Manassus, VA) and cultured in McCoy’s 5A media (Gibco) supplemented with 10% heat-inactivated fetal bovine serum (Gibco), 1X glutaMAX (Gibco) and 1% penicillin/streptomycin (Gibco) and were maintained in the incubator at 37 °C with 5% CO_2_. Cells were seeded on a Starter’s CYTOOchip^TM^ in a 35 mm dish at the density of 75,000 cells/ml, and allowed to adhere for 20 mins before incubation. After 30 mins of incubation, unattached cells were removed by triple rinsing with DPBS (Dulbecco’s Phosphate Buffered Saline). The attached cells were allowed to spread for 3 hours in cell culture media before fixation. Cells were fixed with 4% Paraformaldehyde (Electron Microscopy Science) in PBS (Phosphate Buffered Saline), permeabilized with 0.2% Triton X-100 (Sigma) and stained with Alexa Flour 568 Phalloidin (Life Technologies) overnight. The samples were then mounted on a glass slide with PBS as imaging buffer and sealed by vaseline-lanolin-paraffin mixture [57] for TIRF imaging.

### Total Internal Reflection Fluorescence Microscopy (TIRFM) imaging

The specimens were imaged by Nikon Eclipse Ti-E inverted microscope with motorized total internal reflection fluorescence (TIRF) illuminator. The microscope is equipped with a sCMOS camera (Orca Flash 4.0, Hamamatsu) and a 405/488/561/647 TIRF Laser Dichroic filter (Chroma Technologies). Single cells were acquired under TIRF mode with a 60X oil-immersion objective (NA 1.49 Apo TIRF). Fluorophores were excited at 30% intensity of a 60 mW 561 nm laser.

### Image enhancement by line and orientation filter transform

**Fig. 2:**
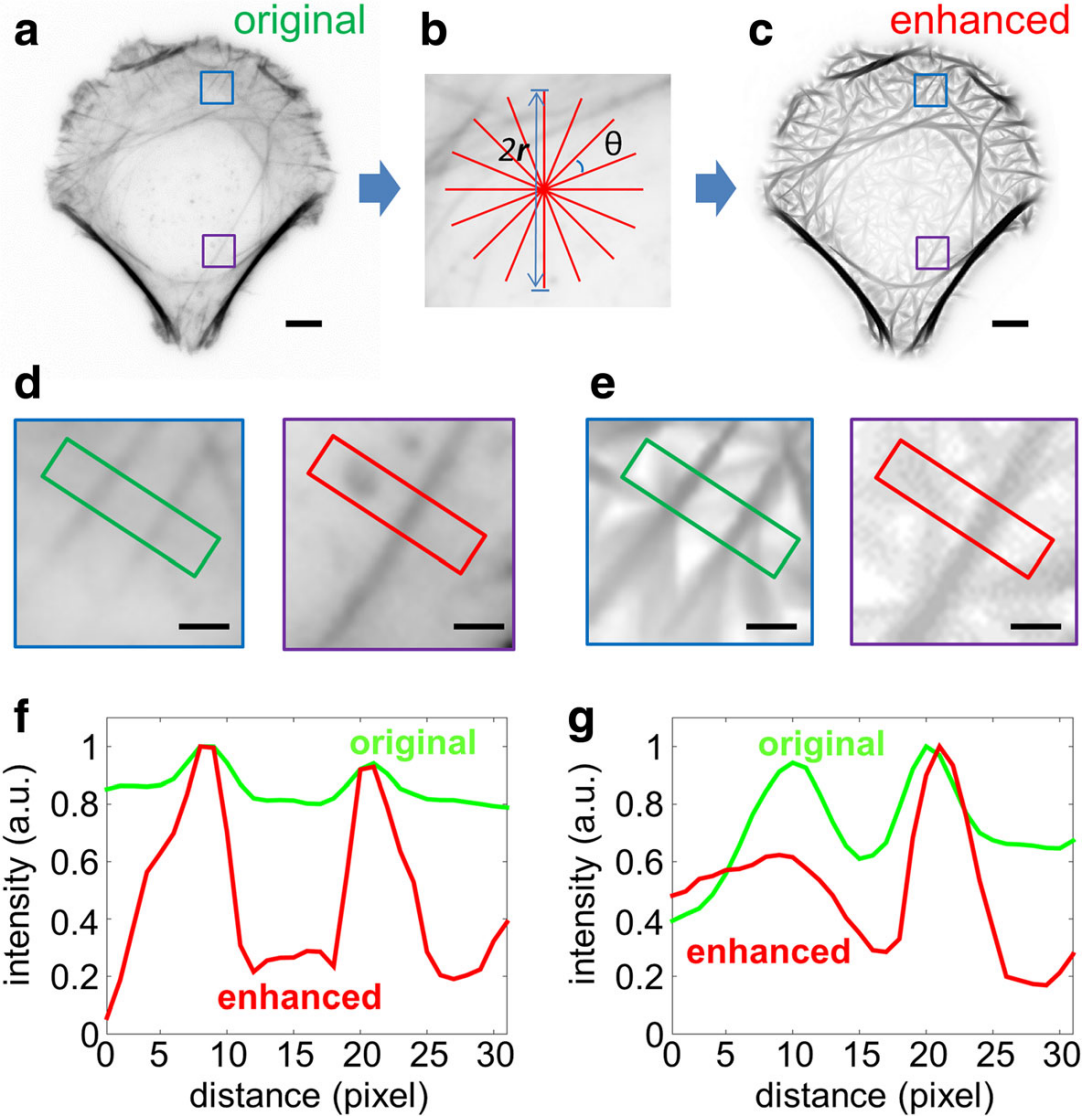
Anisotropic image enhancement. **a**, **c** Images of U2OS cell plated on crossbow-shaped micropattern before (A) and after (C) image enhancement by LFT and OFT (B). Blue box: a region containing two parallel filaments of low contrast with background. Purple box: an area containing a cluster-like noise and a filamentous structure. **b** An illustrative enhancement filter with a total length of 2***r*** and a stepwise rotation angle of ***θ***. Scale bar, 5 μm. **d**, **e** Enlarged images of blue and purple boxes in (A) and (B) respectively. Scale bar, 1 μm. **f** Normalized intensity profiles of the green-cropped regions (D, E, left) the blue square boxes from (A) and (B). **g** Normalized intensity profiles of the red-cropped regions (D, E, right) in the purple square boxes from (A) and (B).

For LFT, at each (***x***, ***y***) pixel we defined a neighborhood of radius ***r***, within which linear features are to be assessed (Fig. 2b). A line segment of length ***2r*** centered at each pixel is rotated stepwise with an angle ***θ*** between -90° and 90° (Fig. 2b). The direction along which the accumulated image intensity is the largest is designated the preferred orientation, ***θ***_***max***_. As defined by Eq. 1 and 2, this process is repeated for all pixels to generate two image maps: the intensity map (*L*_*intensity*_), where each entry is the mean pixel value along the preferential direction of that pixel, and the orientation map (*L*_*orientation*_), which contains the preferred direction at each pixel.

1 $$ {L}_{intensity}\left( x, y\right)=\frac{ma{ x}_{-\frac{\pi}{2}<\theta <\frac{\pi}{2}}\kern0.5em {\displaystyle {\sum}_{t=- r}^r} I\left( x+ tcos\theta, y+ tsin\theta \right)}{2 r+1} $$

2 $$ {L}_{orientation}\left( x, y\right)= arg\left[\frac{ma{ x}_{-\frac{\pi}{2}<\theta <\frac{\pi}{2}}\ {\displaystyle {\sum}_{t=- r}^r} I\left( x+ tcos\theta, y+ tsin\theta \right)\ }{2 r+1}\right] $$

The LFT step above serves to enhance linear features using only the image intensity information. However, in addition to the intensity, linear structures can also be recognized by considering the preferred directions of both the base pixel and its neighbors. To incorporate this information, for each pixel we performed a second filter transform, OFT, to explore whether the neighboring pixels along ***θ***_***max***_ have similar preferential directions. As calculated by Eqs. 3–8, these criteria thus assigned the probability score for a pixel being on a filamentous structure.

3 $$ O\left( x, y\right)={\displaystyle {\sum}_{t=- r}^r\left[{L}_{orientation}\left( x+ t\  cos\ {\alpha}_{max},\  y+ t \sin {\alpha}_{max}\right),\ {\boldsymbol{v}}_{\alpha_{max}}\right]} $$

Where

4 $$ \left[\left(\rho, \theta \right),{\boldsymbol{v}}_{\alpha_{max}}\right]\equiv \rho\ cos\left(2\left(\theta -{\alpha}_{max}\right)\right) $$

5 $$ \rho ={L}_{intensity}\ \left( x, y\right) $$

6 $$ \theta ={L}_{orientation}\left( x, y\right) $$

7 $$ {\boldsymbol{v}}_{\alpha_{max}}= \cos {\alpha}_{max}\ \widehat{\boldsymbol{x}}+ \sin {\alpha}_{max}\ \widehat{\boldsymbol{y}} $$

8 $$ {\alpha}_{max}= \arg\ m a{x}_{-\frac{\pi}{2}<\alpha <\frac{\pi}{2}}\left|{\displaystyle {\sum}_{t=- r}^r\left[{L}_{orientation}\left( x+ t\  cos\ \alpha, y+ t\  sin\ \alpha \right),\ {\boldsymbol{v}}_{\alpha}\right]}\right| $$

### Reconstruction of the stress fiber traces

The significantly increased contrast (Fig. 2f, g) due to LFT and OFT facilitates the use of a segmentation threshold to extract the stress fiber networks. We used Otsu’s method [58] to determine the initial threshold level. Subsequently the binarized image was skeletonized to determine the centerlines of the filaments. However, we noted that a typical stress fiber is rarely an isolated linear structure, but instead is usually associated with numerous small fibrils branching off to the sides (Fig. 1b, c, red, green and blue arrows). This characteristic gives rise to highly branched skeletons, especially in the arc regions, which impedes simple identification of the centerlines of actin bundles.

**Fig. 3:**
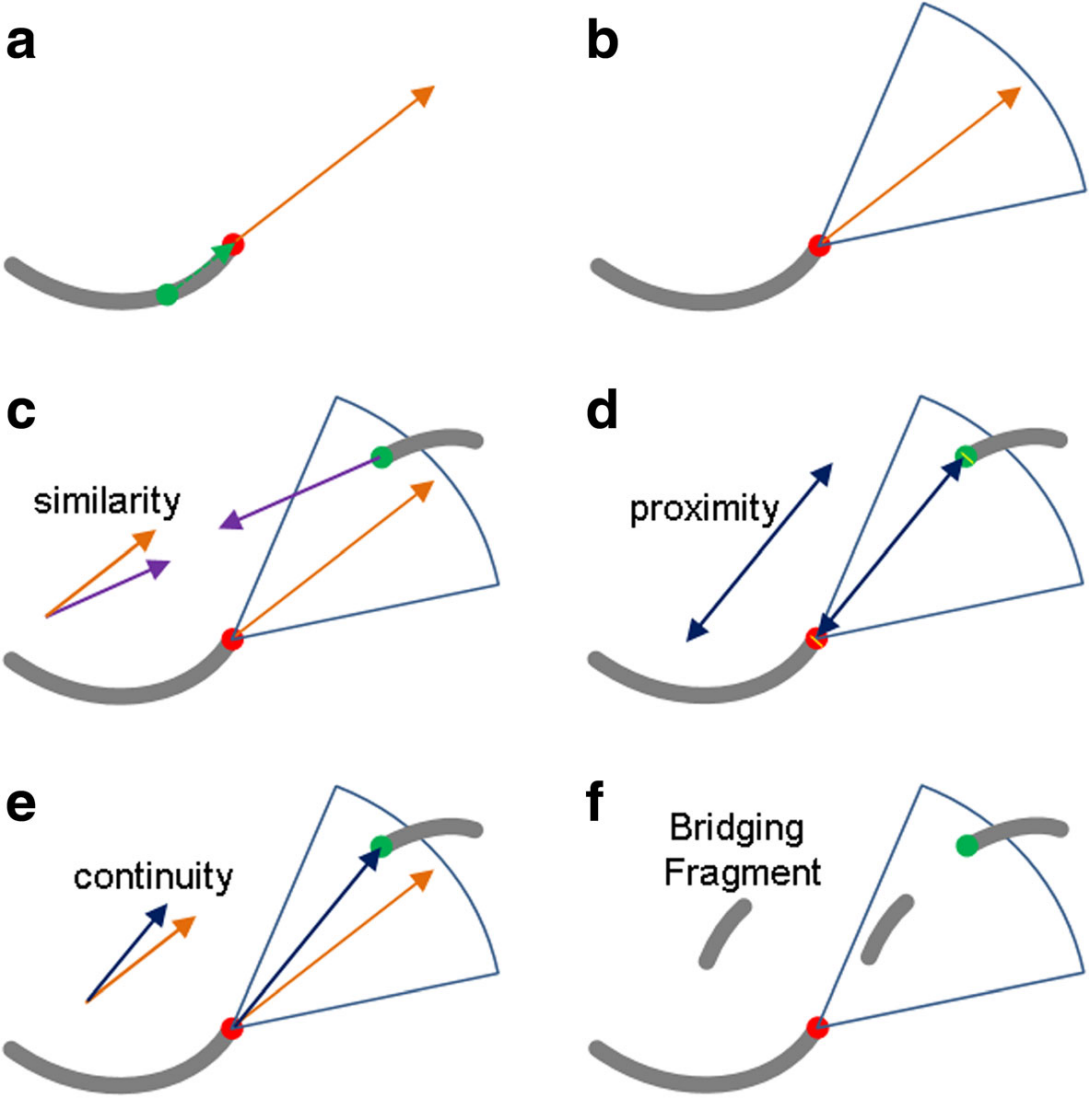
Parameters for fragment termini search and pairing. **a** Definition of the terminus propagation direction: the direction (orange arrow) from the local center of mass (green dot) to the terminus (red dot). **b** Definition of the search sector (blue contour): area swept by the search angle around the propagation direction. **c** Similarity criterion defines the maximum angle difference between the propagation direction (orange arrow) of the based terminus (red dot) and the reverse direction (purple arrow) of the endpoint (green dot) under investigation. **d** Proximity criterion defines the maximum distance (blue two-head arrow) between every two endpoints (red and green dots). **e** Continuity criterion defines the maximum angle difference between the propagation direction (orange arrow) of the based terminus (red dot) and the vector (blue arrow) pointing from the red dot to the green dot. **f** Inner filament criterion for the case where multiple eligible fragments are found. The connection between the red and green termini is to be rejected in preference of the inner filament fragment

To facilitate the tracing of the appropriate stress fiber centerlines, we partitioned the skeleton into separate unbranched linear segments by removing the junction regions whose local 8-connected neighborhood contains more than three filament pixels. This results in a pool of piecewise-linear ‘filament fragments’. Subsequently, we applied a series of geometric constraints to group together the fragments that best capture the stress fibers traces in the original image. We then calculated the propagation direction for each terminus of the fragments, defined as the orientation pointing from the center of mass of the fragment to the tip itself (Fig. 3a). Next, a fan-shaped sector region is generated, anchored at each tip (Fig. 3b). A local search is then performed within each search fan to locate potential tips for fragment connection. Finally, the reconstruction of each stress fiber is carried out by combining every pair of tips, ***i*** and ***j***, that satisfies three geometric constraints: (1) similarity, (2) proximity, (3) continuity, as defined by Eqs. 9, 10, 11 (Fig. 3c–e).

9 $$ \mathrm{Similarity}: D\left({\phi}_{\mathrm{i}},\ {\phi}_{\mathrm{j}}\right)\le {\phi}_{max} $$

10 $$ \mathrm{Proximity}:||{d}_{i, j}||\le {d}_{max} $$

11 $$ \mathrm{Continuity}:{\phi}_i-{\varphi}_{i, j}\le\ {\psi}_{max} $$

with the definition of variables listed in Table 1.

**Table 1:** Definition of geometric constraints parameters

Equation	Symbol	Definition
*9*	*ϕ*_*i*_	Propagation direction of fragment ***i***.
*9*	*ϕ*_*j*_	Propagation direction of fragment ***j***.
*9*	*D*	The function that calculates the orientation difference between ϕ_*i*_ and the reverse direction of ϕ_*j*_.
*9*	*ϕ*_*max*_	The maximum allowable difference between ϕ_*i*_ and the reverse direction of ϕ_*j*_.
*10*	*d*_*i*,*j*_	The magnitude of the distance vector between ***i*** and ***j***.
10	*d*_*max*_	The maximum allowable gap length.
11	*φ*_*i*,*j*_	The direction of the distance vector pointing from ***i*** to ***j***.
11	*ψ*_*max*_	The maximum allowed direction mismatch between the fragment ***i*** and distance vector.
12	*ϕ*_*m*_^*a*^	The propagation direction of the tip of bridging fragment ***m*** close to fragment ***i***.
13	*φ*_*i*,*m*_^*a*^	The distance vector between fragment ***i*** and the its closest tip of fragment ***m***.
14	*ϕ*_*m*_^*b*^	The propagation direction of the tip of bridging fragment ***m*** close to fragment ***j***.
15	*φ*_*j*,*m*_^*b*^	The distance vector between fragment ***j*** and the its closest tip of fragment ***m***.
16	*C*_*i*_	Eligibility cost for connecting the base terminus and its ***i***th partner tips.
16	*C*_*angle*_*weight*_	Weight for similarity criterion.
16	*C*_*gap*_*weight*_	Weight for continuity criterion.
16	*Δ* *θ*_*i*_	Difference for similarity.
16	*Δ* *θ*_*gapi*_	Difference for continuity.

However, in many occasions, one or more bridging fragments that themselves satisfying these three criteria can be found in the search fan (Fig. 3f). To address this, the fourth criterion is applied to check for the existence of such inner fragments and whether they are eligible to bridge the two fragments being investigated, defined as follows (variables definition in Table 1):

12 $$ D\left({\phi}_i,\ {\phi}_m^a\right)\le {\phi}_{m ax} $$

13 $$ {\phi}_i-{\phi}_{i,\  m}^a\le {\psi}_{max} $$

14 $$ D\left({\phi}_j,\ {\phi}_m^b\right)\le {\phi}_{m ax} $$

15 $$ {\phi}_j-{\phi}_{j,\  m}^b\le {\psi}_{max} $$

For each iteration, it is possible that multiple fragment termini may satisfy the aforementioned four conditions with respect to the base terminus. To select the optimal partner terminus for combination, we therefore introduced a scoring system to compute the priority of all termini based on how they satisfy the similarity and continuity criteria as shown in Eq. 16 (variables definition in Table 1).

16 $$ {C}_i = {C}_{angle\_ weight}\times \frac{\varDelta {\theta}_i}{max\left(\varDelta {\theta}_1,\varDelta {\theta}_2,\varDelta {\theta}_3,\dots, \varDelta {\theta}_n\right)}+{C}_{gap\_ weight}\times \frac{\varDelta {\theta}_{gap i}}{max\left(\varDelta {\theta}_{gap1},\varDelta {\theta}_{gap2},\varDelta {\theta}_{gap3},\dots, \varDelta {\theta}_{gap n}\right)} $$

The termini pair with the lowest score are then assigned to the same stress fibers, while the unpaired fragment termini are designated as the termini of stress fibers. The process is iterated for all fragments and, when completed, yields the most probable centerline trace of each stress fiber within the networks. Using a desktop workstation, the entire calculation for a typical image (512 × 512 pixels or 55 × 55 μm) was completed in less than two minutes. Subsequently, the centerline trace for each stress fiber can be used for further analysis of the stress fibers in lieu of the entire image, thereby helping to reduce the data set dimension. For example, the orientation of the stress fibers can be calculated using the local information centered around each pixel along the centerline.

### Generation of synthetic images and quantification of extraction accuracy

**Fig. 4:**
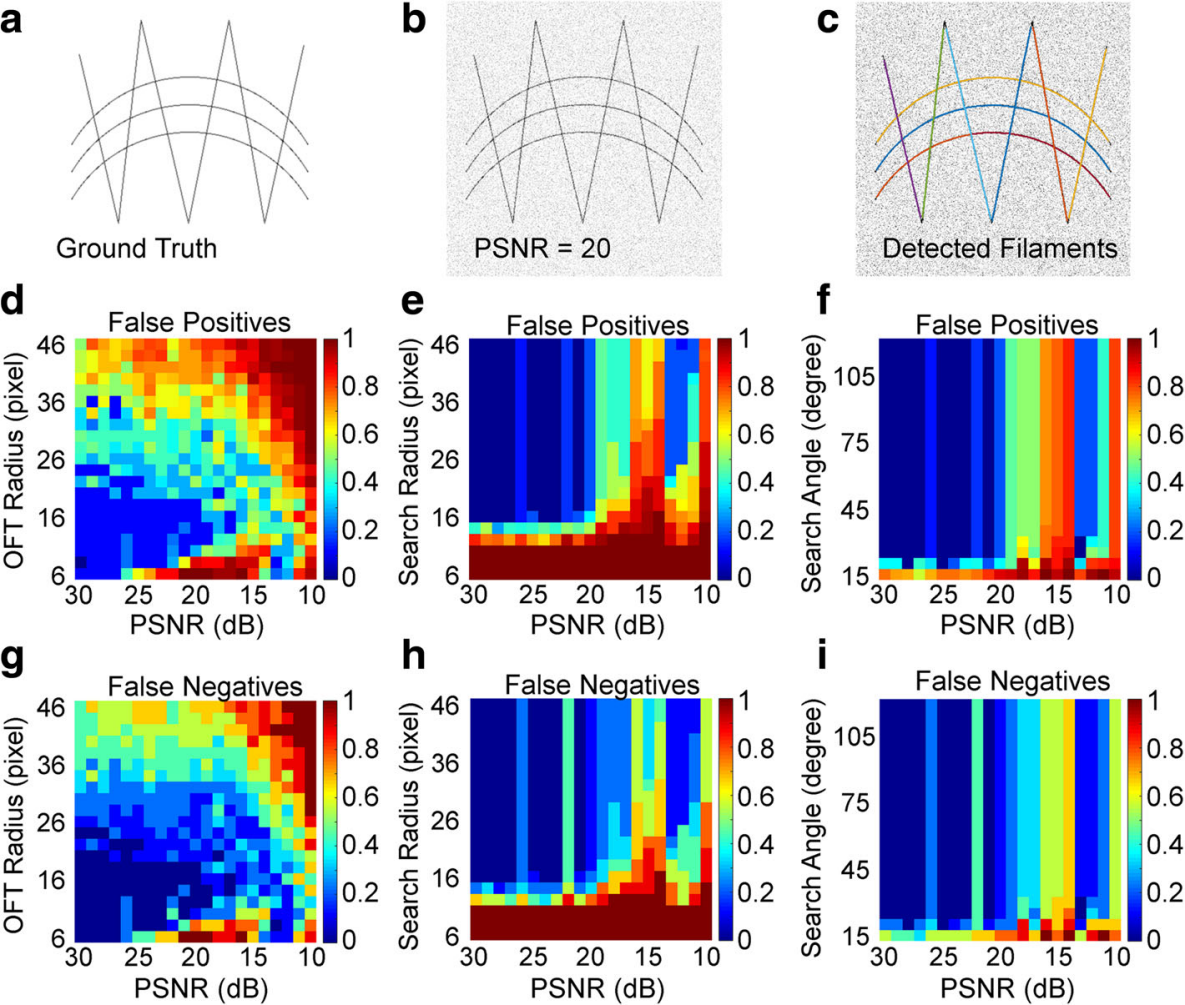
Assessment of filament reconstruction accuracy. **a** Synthetic ground truth image containing both curved and straight lines, mimicking stereotypical stress fiber arrangement. **b** Synthetic image with PSNR of 20 dB. **c** 9 Detected filaments shown in different colors from (B). **d**-**i**) Maps of False positives (D-F) and false negatives (G-I) for the accuracy of individual filaments detection (described in text) are computed as a function of image noise (PSNR) versus filter radius (D, G), search radius (E, H), and search angle (F, I), respectively. Pixel size for synthetic images, 108 nm

To systematically evaluate the capability of our method in extracting stress fiber filaments, sensitivity analyses were performed to benchmark the dependence of our method on the values of key parameters and the magnitude of background noise. As shown in Fig. 4a, we generated synthetic images using simple geometric patterns of curves and straight lines as the ground-truth, approximating the two types of stress fibers shapes commonly observed in cells. The ground truth skeletons were convoluted with a Gaussian filter with a width comparable to the resolving power of our TIRF microscope (σ = 72 nm, image pixel size 108 nm), and corrupted by Gaussian noise added at varying Peak Signal-to-Noise Ratio (PSNR) (Fig. 4b, Additional file 4: Figure S2). To quantify the performance of our method in detecting individual fibers (Fig. 4c), we determined the false positives and false negatives ratios, defined as 1-(M/D) and 1-(M/G), respectively, where D is the number of computer-identified filaments, G is the number of ground truth filaments and M denotes the number of matches between D and G [59].

### Automated determination of stress fiber widths

The widths of the stress fibers are known to be strongly correlated with both cell contractility or activities of actin regulatory pathways [1, 23, 60–62], and thus can serve as a useful read-out of cell mechanical properties. Although this can be calculated interactively from line profile of stress fibers, due to the large numbers of stress fibers per cells, we sought to automate this process. For each thick stress fiber trace (Additional file 5: Figure S3A, synthetic image example shown), we first calculated the Euclidean distance map (Figure S3D, synthetic image example shown). Since each stress fiber can be considered as an open curve, each distance level generated by connecting pixels with the same distance value appears as a closed loop enveloping the stress fiber. Based on the original fluorescence micrographs, we then calculated the mean of the gradients along each distance level. The mean gradients as a function of the distances from the stress fibers were then obtained, and the distance level with the highest mean image gradient is identified, with the average width of the stress fiber defined as twice this value (Additional file 5: Figure S3G-J).

### Extracting information from secondary actin stress fiber networks

**Fig. 5:**
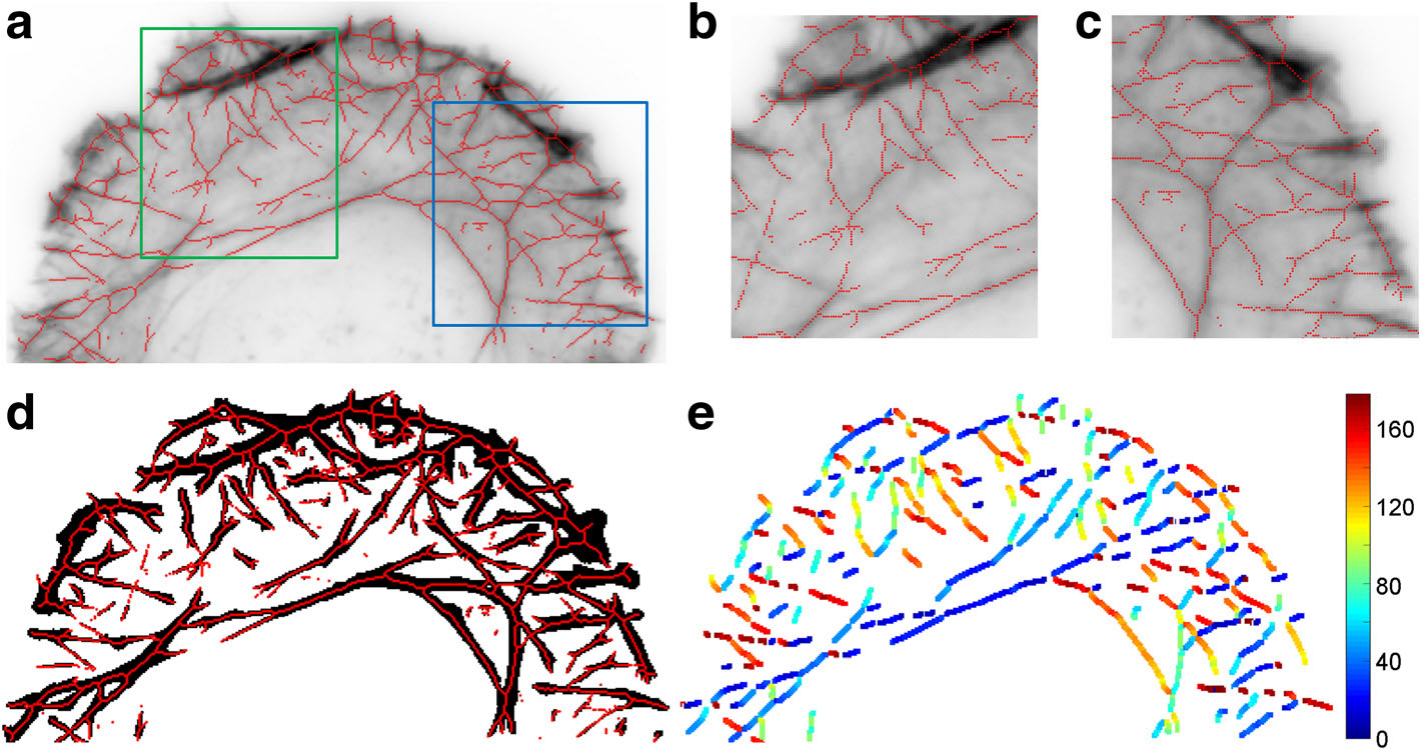
Stress fiber extraction and orientations. **a** Skeleton of stress fibers (red) overlaid on TRFM image of the protrusive region of U2OS cell plated on crossbow-shaped micropattern. **b**, **c** Enlarged regions of green and blue boxes in (A). **d** Binary image of stress fiber network overlaid with network skeleton (red). **e** Distribution of actin orientations. Colorbar, degree

The actin stress fibers exhibit significant variations in their sizes and intensity. Thus, while the prominent primary stress fibers can be easily detected, lower intensity secondary fibers that often form complex networks are more challenging to segment accurately (Fig. 5a-c). Nevertheless, as these types of stress fibers can comprise a significant portion of F-actin structures in cells, we also implemented a method to glean quantitative information from such networks. Following the extraction of the high-intensity stress fiber networks from the cell images, Otsu thresholding is used to segment cell areas devoid of thick stress fibers (Fig. 5a-c). The integrated intensity of pixels within this region can then be calculated, for example, as a function of the distance from the cell edge (Fig. 5d, black region). This provides a useful metric for the density distribution of the actin networks as a function of the cell morphology, particularly in case of cells on micropattern, described further below, where the cell edge can be used as a common spatial frame of reference.

### Statistical analysis and computation

The computational routines were programmed in MATLAB (Release R2015a, Natick, MA). All computations were performed on Windows 7 workstation (Intel(R) Xeon(R) CPU E5-2640 v3 @ 2.60GHz; RAM 192GB; 64-bit OS). Statistics and graphing were performed in MATLAB.

## Results and Discussion

### Fluorescence imaging and analysis of actin stress fibers

The spatial distribution of the actin stress fibers is closely coupled to the morphology of the cells. However, with conventional cell culture methods, cell shapes for a given population become highly heterogeneous, complicating a systematic study of the spatial organization of actin structures. One of the methods to regularize cell morphology is the use of micropatterns to constrain cell adhesion to within areas printed with extracellular matrix proteins such as fibronectin (insets 1-3 in Fig. 1b), with the outlying areas coated with non-adhesive materials [31, 63, 64]. As seen in Fig. 1b, human osteosarcoma (U2OS) cells cultured on such fibronectin-micropatterned cover glasses (CYTOOChips, Cytoo Inc.) were highly restricted to geometric forms such as Y-, crossbow-, and disc-shaped patterns, exhibiting significant uniformity across the population (Fig. 1b, Additional file 1: Figure S1). Using Alexa Fluor 568 phalloidin to label F-actin, diffraction-limited fluorescence images of stress fiber organization can be obtained by total internal reflection fluorescence microscopy (TIRFM) imaging.

As seen in Fig. 1b-c and Additional file 1: Figure S1, while the stress fibers are often the most prominent F-actin containing features, fluorescence micrographs of F-actin usually contain numerous other image features that pose significant challenges for the automated extraction of stress fibers. These include the complex organization of the filaments, such as filament intersection and convergence (Fig. 1b, c, Additional file 1: Figure S1), numerous F-actin-containing cellular structures which may appear as bright puncta (Fig. 1b, c, Additional file 1: Figure S1), and nebulous background intensity which reduces the local contrast of the stress fibers (Fig. 1b, c, Additional file 1: Figure S1). Thus, while the qualitative patterns of stress fiber organization can be readily recognized by visual inspection, the automated extraction of the fibers against feature clutters and high background noises remains a difficult task. To address these, we made use of anisotropic image enhancement methods to accentuate the fibrous structure of interest. Anisotropic image enhancement has been widely used for recognizing cellular structures such as cytoskeleton and membrane, with prior knowledge about the shapes of interest [56, 65–67]. We found that robust anisotropic enhancement can be achieved using the LFT and OFT methods which are relatively easier to implement compared to other approaches. LFT and OFT electively highlight all component pixels along filamentous structures, while suppressing non-linear features [56]. As shown in Fig. 2, LFT and OFT enhance the relative contrast of filamentous features and also suppress the intensity of non-filamentous structures, therefore enabling the use of a simple binarization threshold to extract features of interest. The binarized image of the stress fiber networks is then skeletonized and regions of filament junctions are removed to generate unbranched linear fragments. Subsequently, the filaments are reconstructed by using four geometric criteria for filament fragments recombination, as illustrated in Fig. 3. These computational steps, and subsequent quantitative analysis of the networks, can be performed using the included software package, SFEX (Stress Fibers Extractor), described in the Supplementary Information.

### Assessing the performance of stress fiber reconstruction

Since the architectures of stress fiber networks differ drastically between different cells even under the same condition, the optimal parameter set for fiber extraction also varies from cell to cell. To aid users in estimating the appropriate input parameter range, particularly against image noise, we performed sensitivity analyses by systematically varying a given pair of key parameters, keeping the rest fixed, and used SFEX to extract the stress fibers from synthetic images as shown in Fig. 4a–c. The results of the analyses were then scored against the ground-truth and the errors were visualized as the heat maps shown in Fig. 4d–i.

We first evaluated how the neighborhood radius ***r*** of the LFT/OFT enhancement together with the noise level of the original images affected the accuracy of stress fiber detection. As shown in Fig. 4d and g, both the neighborhood radius ***r*** and image quality are strong determinants of the tracing accuracy. The highest accuracy (low false positives and false negatives ratio, blue regions in Fig. 4d and g) was found to correspond to ***r*** = ~1.2 μm, which represents the optimal balance, as the large values of ***r*** may introduce distortion to curved fibers and hence a low tracing accuracy, while the smaller values of ***r*** may not provide sufficient enhancement for the fibers relative to noises. Also, these results suggest that our method appear to perform reliably for image quality exceeding PSNR ~ 20, with rapid deterioration with the increase in image noise (see Additional file 4: Figure S2 for examples of noise levels).

We next explored how the search radius parameter for the fragment reconstruction affected fiber reconstruction accuracy. Surprisingly, we observed that beyond the lower limit of search radius ~16 pixels which corresponds to ~1.7 μm, a relatively wide range of search radius can be used to obtain high reconstruction accuracy, as seen in Fig. 4e and h. This permissiveness suggests that the detection accuracy is not significantly affected as long as the search region encloses enough potential fragment termini for recombination analysis. Also this indicates that our algorithm for termini pairing is probably sufficiently robust that the optimal pairing can be determined regardless of the size of the search fan. Likewise, the performance of our method is relatively resilient with respect to the search fan angle beyond ~20 degree, as shown in Fig. 4f and i, suggesting that a relatively large search sector should be used to avoid potential mismatch of termini pair. Similar to the case of the OFT radius above, the accuracy degrades rapidly once image quality is reduced below PSNR ~20. Here, the filament extraction error likely originates from noise-induced error in the filament propagation direction (Fig. 3b). Interestingly, we note that a narrow region of relatively good accuracy can be observed at ~ PSNR 12-14. As can be seen from Additional file 4: Figure S2, the images are significantly degraded by noise in that range, such that the filaments are extracted in multiple small segments. In this case, the bridging filament criterion (Fig. 3f) would be invoked, which may help improve the accuracy to a certain extent. However, as the zone where this effect takes place is relatively narrow, it is advisable to obtain image quality exceeding PSNR ~ 20 for accurate analysis of the stress fibers.

We also performed sensitivity analysis for the stress fiber width determination. For this, we used a ground truth image consisting of a curved fiber (Additional file 5: Figure S3A and Additional file 6: Figure S4A). The synthetic images were corrupted by noise (Additional file 6: Figure S4B-G) and the width of the filaments calculated using the distance map-based method described above. As shown in Additional file 5: Figure S3B-C, E-F, the width of the fiber obtained from the analysis appears to capture well the width of the fiber across a wide range of noise level, (Additional file 6: Figure S4H), suggesting that our distance map gradient method is highly resistant to image noise.

### Quantitative analysis of geometrically confined cells

**Fig. 6:**
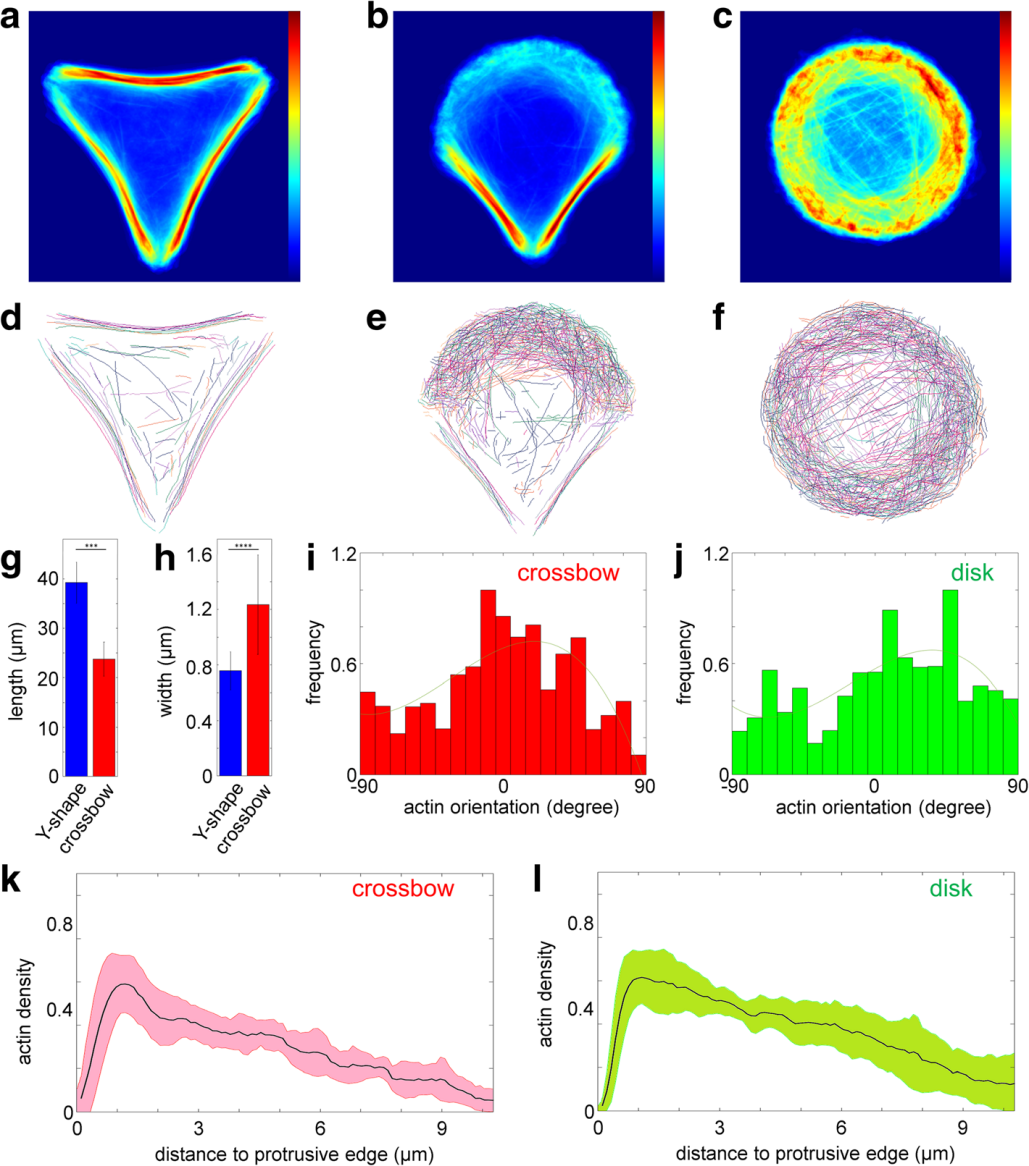
Extraction and quantitative analysis of stress fibers network. **a**–**c** Overlay of aligned U2OS cells plated on Y- (A), crossbow- (B) and disc-shaped (C) micropatterns. Colorbar, intensity. **d**–**f**) Overlay of detected stress fibers from aligned U2OS cells plated on Y- (D), crossbow- (E) and disc-shaped (F) micropatterns. Filaments with the same colors are from the same cell. **g**, **h** Comparision of the length (G) and width (H) of ventral stress fibers in cells plated on Y- (A) and crossbow-shaped (B) micropatterns. ***: *p* < 2x10^-21^, t-test. ****: *p* < 5x10^-8^, t-test. **I**, **J**) Histograms of F-actin orientations in protrusive regions of cells plated on crossbow- (I) and disc-shaped (J) micropatterns. Green lines: 3rd order polynomial fit. **k**, **l** Normalized actin density as a function of distance levels in cells seeded on crossbow- (K) and disc-shaped (L) micropatterns. Light red and green bands depict their standard deviations. *n* = 10 cells for each micropattern

In recent years, image-based morphometric profiling of cells has been an active area of investigation both in basic cell biological studies and in theranostic developments [68, 69]. In particular, the invention of extracellular matrix micropatterning technologies which standardize cell shapes [31, 63, 64] has greatly facilitated image processing and statistical analysis. Fluorescence micrographs of numerous cells with similar geometrically well-defined shapes can be rapidly acquired by automated microscopes, and ensemble-averaged to yield maps of how specific molecules are stereotypically organized [63, 70, 71]. However, the effect of shape confinement on actin stress fibers organization has not been systematically investigated, despite their dramatic differences between cell shapes (Fig. 6a-f, Additional file 1: Figure S1), in part due to the difficulties of extracting stress fibers from the images. Thus in this study we used our method to explore how the micropatterns may influence actin stress fibers organization.

For this work, we made use of three different micropatterns which are designed to elicit different cell morphological responses, but with comparable total cell areas: Y-shaped, which promotes strong adhesion and prominent stress fibers at cell edges spanning between each vertex (Fig. 1b, left); disc-shaped, which keeps the cells unpolarized, thus devoid of thick stress fibers (Fig. 1b, right), featuring instead circumferential network of secondary stress fibers that connects with adhesion sites near the cell boundary at semi-regular intervals; and crossbow-shaped, which induces clear front-back polarization, with prominent stress fibers connecting the vertices, converging on the cell rear, and intricate networks of secondary actin stress fibers in the frontal arc (Fig. 1b, middle).

To overlay actin images of cells on a given micropattern, we segmented the cells and determined the centers of mass (COMs) of the cell region (Additional file 7: Figure S5A-C), and then performed translation and rotation to align their COMs (Additional file 7: Figure S5D-F). The ensemble image is then calculated from the mean of the aligned images. As shown in Fig. 6a, b, d, e, we observed that the prominent primary stress fibers appear to be nearly uniform between different cells on similar patterns, while the organization of secondary actin stress fibers, especially toward the cell front in polarized cell, is much more complicated. Thus, although micropatterns help standardize the overall cell morphology, significant cell-to-cell variation in discrete structures such as actin stress fibers are still observed and thus spatial information on stress fibers organization is significantly obscured by simple ensemble-averaging analysis.

### Architecture of actin stress fiber networks as a function of cell shapes

Since the architecture of the stress fiber networks can be analyzed on a cell-to-cell basis using SFEX, we next demonstrated the utility of our approach in quantifying actin stress fibers in cells on various micropatterns. We performed stress fiber analysis on these cells and outlined below some important metrics useful for differentiating between their distinct actin stress fiber organization phenotypes.

Stress fiber networks, especially in protrusive regions, play important roles in cell polarity and migration. The mesh of secondary actin stress fibers forms ‘band’- like lamella structure [21, 72] parallel to the protrusive edges of the cell (Fig. 6b, c, e, f), which are believed to be the zones of contractility generation by myosin II motors. We therefore first investigated whether the size of the lamella zone is dependent on micropattern-induced cell polarity. We compared actin organization in unpolarized disc pattern and polarized crossbow pattern, plotting the density of F-actin (coverage ratio of F-actin versus total cell area) as a function of the distance from cell edge, using the curved edge of the cell as the spatial frame of reference. As shown in Fig. 6k-l, F-actin densities peak at distance ~1 μm behind the cell edge, then monotonously decrease toward the cell interior. A similar pattern is observed for both unpolarized (disc) and polarized (crossbow), suggesting that the overall radial organization of the actin cytoskeleton may be comparable, regardless of the extent of cell polarization.

We next investigated the orientation distribution of individual secondary actin stress fibers, as these may be another useful indicator of network organization. We skeletonized the network and removed the junction regions to generate linearized fragments and calculated the orientation of F-actin for each skeleton pixel using a 15-by-15-pixel window (Fig. 5e). Our analysis revealed a non-uniform arrangement of actin orientations in polarized cells (cross-bow) as shown in Fig. 6i relative to non-polarized (disc) cells (Fig. 6j, *p* = 8.4 x 10^-5^, Kolmogorov-Smirnov test). Such difference in the azimuthal organization of the actin networks likely reflects the shape constraint imposed by the cell edge, since such secondary actin stress fibers are believed to arise from the lamellipodial front (i.e. the arc) [73], and thus may originate uniformly in disc-shaped cells, but with orientation preference in crossbow-shaped cells. Thus, while the actin networks exhibit comparable radial organization, polarity appears to manifest in terms of azimuthal organizational bias. Future studies that systematically vary the dimension or the symmetry of the pattern may thus be useful in studying how mechanical constraints are sensed and propagated into actin organization differences and vice versa.

Finally, we note that while the width of the stress fibers are generally considered to be mechanosensitive, dependent on factors such as substrate stiffness as well as myosin-II dependent contractility [1, 23, 60–62], such relationship is thus far considered to be largely qualitative [23, 62], and a predictive relationship between stress fiber attributes and cell mechanics has not been established. Towards addressing this question, we explored whether the dimensions of the thick stress fibers are dependent on the size of non-adhesive regions in micropatterns, that is, their lengths. As shown in Fig. 6a, b, actin filament forms thick bundles spanning the entire borders of the non-adhesive regions. The average total length of stress fibers on Y-shaped pattern is ~1.6 times longer than those on crossbow-shaped pattern (Fig. 6g, *p* < 2x10^-21^, t-test). Interestingly, the width of the stress fibers on the latter is also ~1.6 times larger than on the former (Fig. 6h, *p* < 5x10^-8^, T test) indicative of an inverse proportional relationship between the width and length of stress fibers, which may be interesting to investigate further by variation of the pattern size and in conjunction with pharmacological perturbations.

## Conclusion

We proposed an integrated computational strategy for the enhancement, detection, and analysis of actin stress fibers from fluorescence images of F-actin in adherent tissue culture cells. The associated software package, SFEX, is an open-source and highly customizable tool equipped with graphical user interface. We implemented anisotropic neighborhood-based filtering to enhance linear features corresponding to the stress fibers relative to other undesirable structures. This enables efficient segmentation and skeletonization of stress fibers to yield the centerline traces. Using a set of geometric criteria, the stress fiber skeletons were regrouped from piecewise-linear fragments to reconstruct the entire networks. We evaluated the robustness of our approach and defined the parameter range for optimal performance. Using cells cultured on well-defined micropattern substrates, we analyzed their stress fiber organization to highlight their dependence on cell shapes. Our approach can be performed with minimal user input and thus should be amenable for processing large datasets. This could be useful both for time-lapse movies where the analysis of large number of frames by self-consistent criteria are required, or for high-throughput applications whereby cells are subject to external mechanical or biochemical perturbations.

## Additional files

Additional file 1: Figure S1. Actin stress fibers architectures of U2OS cells on micropatterns. Inverse contrast TIRFM images of F-actin in U2OS cells plated on Y- (top row), disk- (middle row) and crossbow-shaped (bottom row) micropatterns. Scale bar, 5 μm (TIF 4029 kb).

Additional file 2: SupplementarySoftware_SFEX_BINF-D-16-00942. Stress Fiber Extractor (SFEX) 1.0. This file contains a test image and all the source code associated with the method proposed in this study (DOCX 6571 kb).

Additional file 3: SupplementarySoftware_SFEX User-Manual-BINF-D-16-00942. SFEX 1.0 User Manual. This file is a step-by-step introduction about the usage of SFEX (The software in Additional file 2) (ZIP 633 kb).

Additional file 4: Figure S2. Synthetic images used for assessing filament reconstruction accuracy. Synthetic ground truth images with introduced noise at different levels (TIF 6847 kb).

Additional file 5: Figure S3. Sensitivity analysis of filament width measurement. A) Ground truth image. B, C) 3D visualization of images with low (B) and high (C) noise. Projected 2D images are shown below their 3D view. Detected filament contour is highlighted in black. D) Distance map based on ground truth image (A). Colorbar, distance to the centerline of ground truth filament. E, F) Enlarged view of regions highlighted by blue arrows in (B) and (C). Detected filament contour is highlighted in black. G) Stress fibers in a U2OS cell plated on Y-shaped micropattern. Contours of ventral stress fibers are highlighted in red. Scale bar, 5 μm. H-J) Enlarged views of regions indicated by red arrows in (G). Scale bar, 1 μm (TIF 13861 kb).

Additional file 6: Figure S4. Images for sensitivity analysis of filament width measurement. A) Synthetic ground truth image. B-G) Synthetic image with introduced noise at different levels. H) Detected filament length as a function of image noise (TIF 4838 kb).

Additional file 7: Figure S5. Alignment of cells on micropatterns. A-C) Overlay of cell contours before alignment for Y, crossbow, and disc patterns, respectively. Crosses denote the center-of-mass (COM) of the cell regions. D-F) COM-based overlay of aligned cell contours. *n* = 10 for each micropattern (TIF 943 kb).

## Abbreviations

TIRFM: Total Internal Reflection Fluorescence Microscope
F-actin: Filamentous Actin
LFT: Line Filter Transform
OFT: Orientation Filter Transform
SFEX: Stress Fiber Extractor
U2OS: Human Osteosarcoma Cells
DPBS: Dulbecco’s Phosphate Buffered Saline
PBS: Phosphate Buffered Saline
PSNR: Peak Signal-to-Noise Ratio
COM(s): center(s) of mass
