# Supplementary figures and images for: An integrated enhancement and reconstruction strategy for the quantitative extraction of actin stress fibers from fluorescence micrographs
Source: BMC Bioinformatics. 2017 May 22;18:268. doi: 10.1186/s12859-017-1684-y (PMC5440974; doi:10.1186/s12859-017-1684-y)

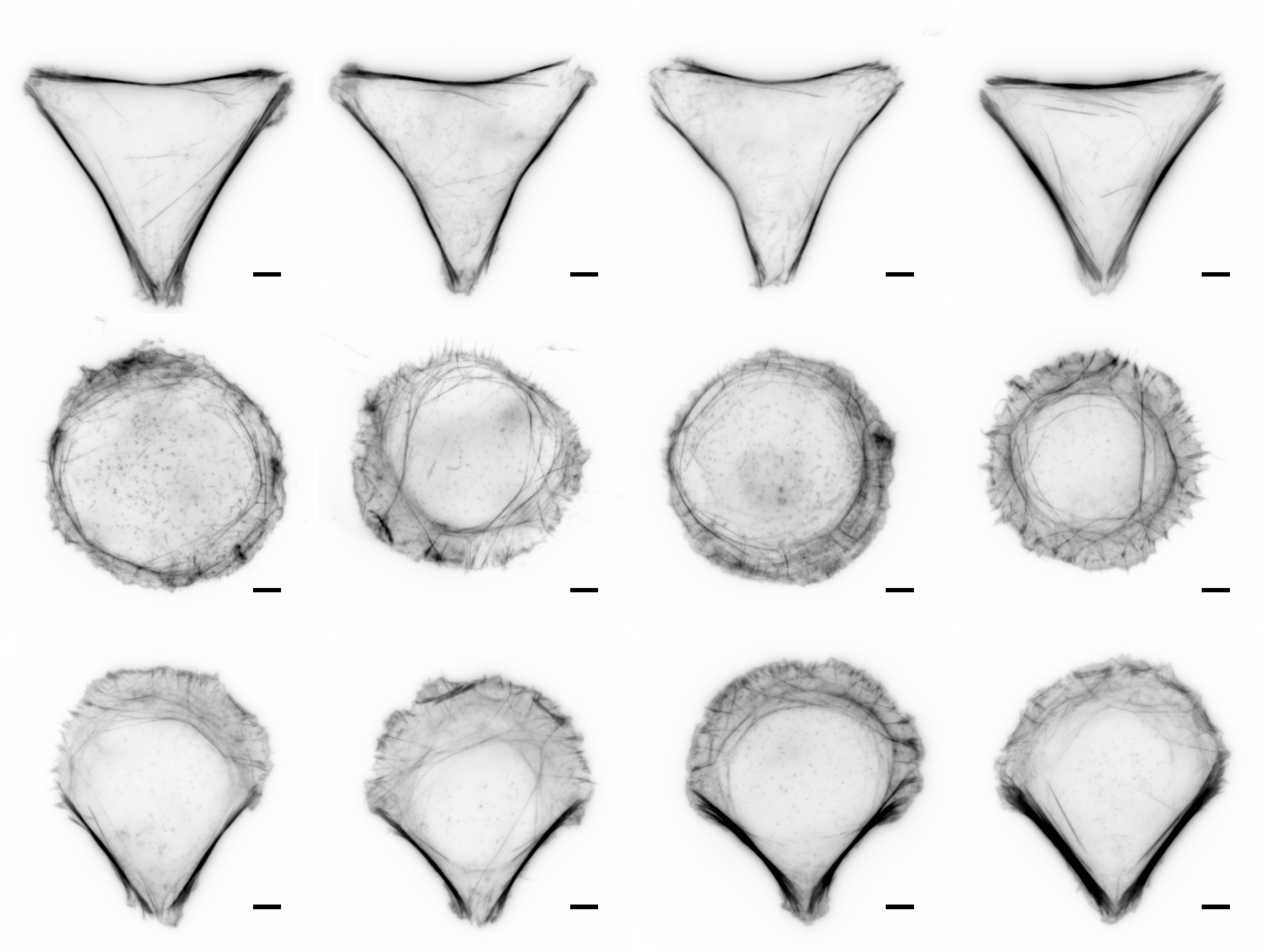

Supplement: Supplementary file 1 — Actin stress fibers architectures of U2OS cells on micropatterns. Inverse contrast TIRFM images of F-actin in U2OS cells plated on Y- (top row), disk- (middle row) and crossbow-shaped (bottom row) micropatterns. Scale bar, 5 μm (TIF 4029 kb). [file 12859_2017_1684_MOESM1_ESM.tif]

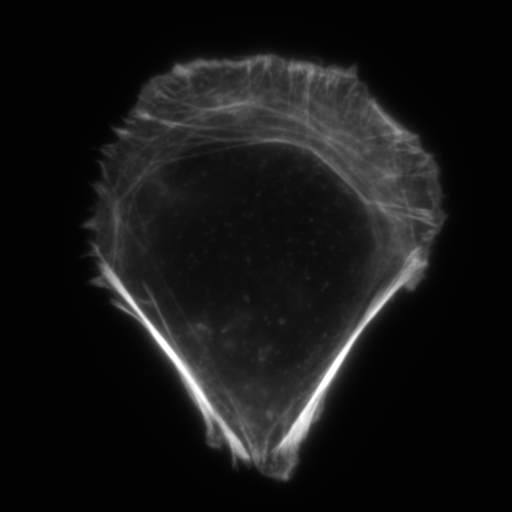

Supplement: Supplementary file 3 — SupplementarySoftware_SFEX User-Manual-BINF-D-16-00942. SFEX 1.0 User Manual. This file is a step-by-step introduction about the usage of SFEX (The software in Additional file 2) (ZIP 633 kb). [file 12859_2017_1684_MOESM3_ESM.zip › SupplementarySoftware_SFEX_BINF-D-16-00942/Crossbow.tif]

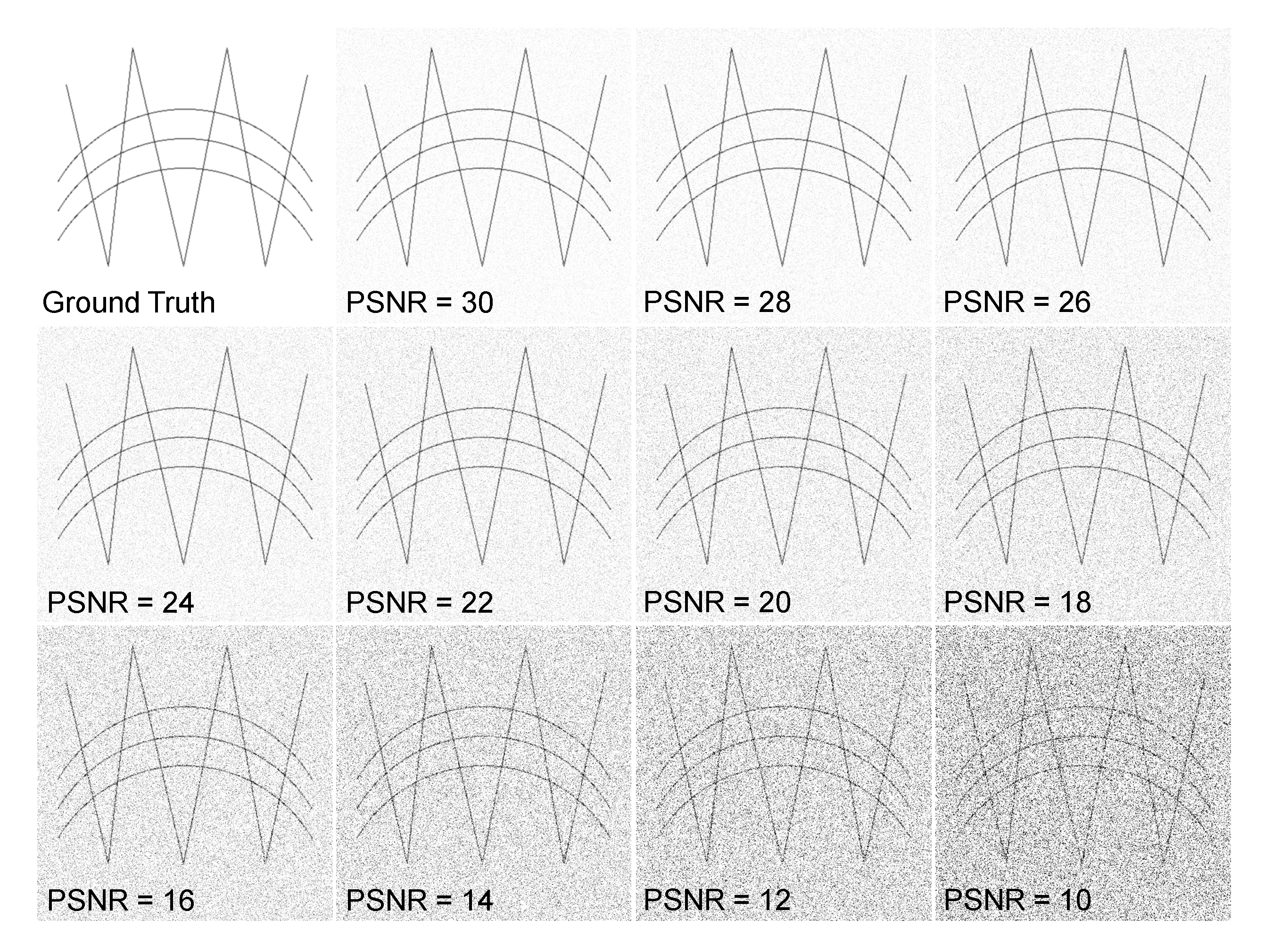

Supplement: Supplementary file 4 — Synthetic images used for assessing filament reconstruction accuracy. Synthetic ground truth images with introduced noise at different levels (TIF 6847 kb). [file 12859_2017_1684_MOESM4_ESM.tif]

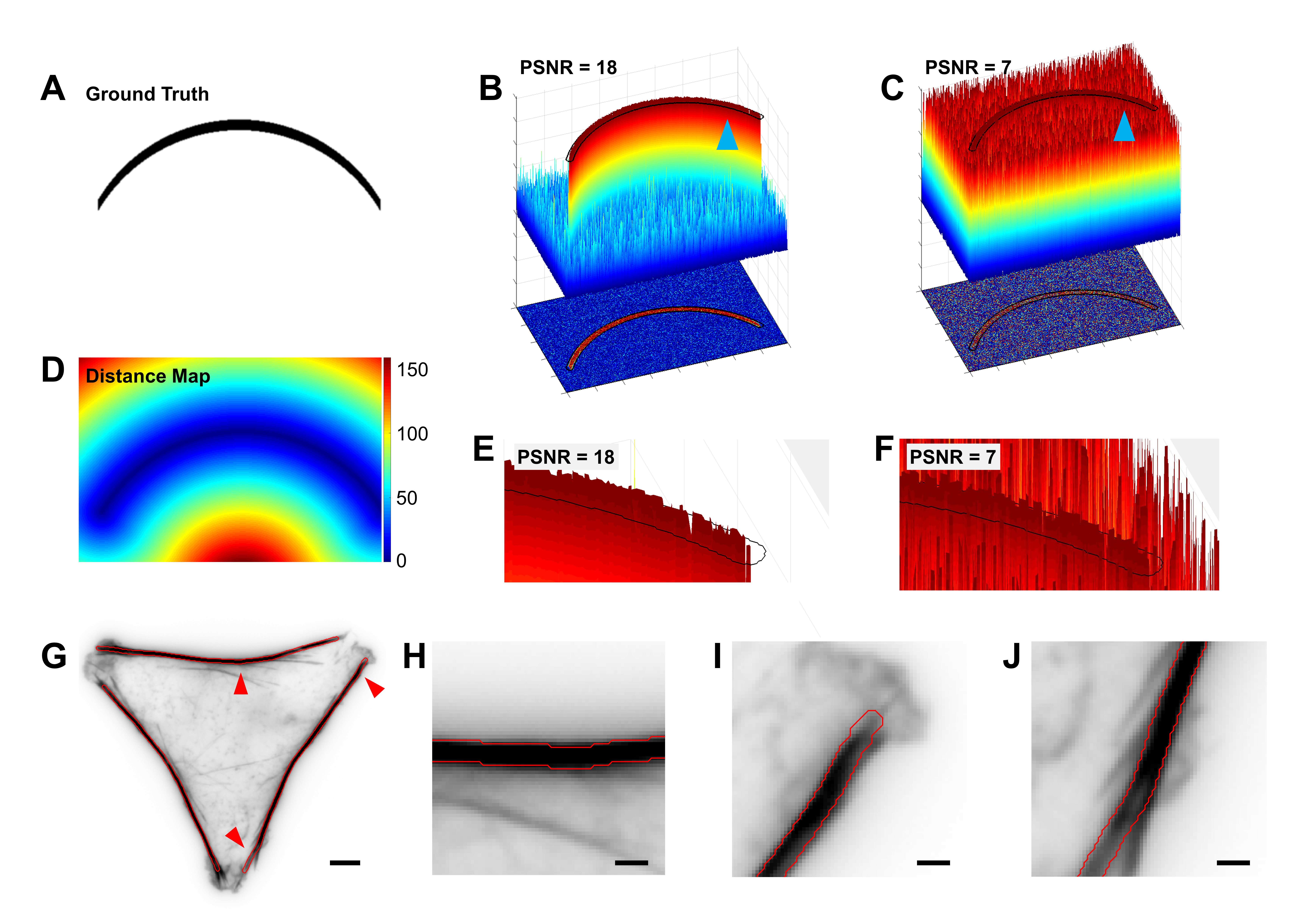

Supplement: Supplementary file 5 — Sensitivity analysis of filament width measurement. A) Ground truth image. B, C) 3D visualization of images with low (B) and high (C) noise. Projected 2D images are shown below their 3D view. Detected filament contour is highlighted in black. D) Distance map based on ground truth image (A). Colorbar, distance to the centerline of ground truth filament. E, F) Enlarged view of regions highlighted by blue arrows in (B) and (C). Detected filament contour is highlighted in black. G) Stress fibers in a U2OS cell plated on Y-shaped micropattern. Contours of ventral stress fibers are highlighted in red. Scale bar, 5 μm. H-J) Enlarged views of regions indicated by red arrows in (G). Scale bar, 1 μm (TIF 13861 kb). [file 12859_2017_1684_MOESM5_ESM.tif]

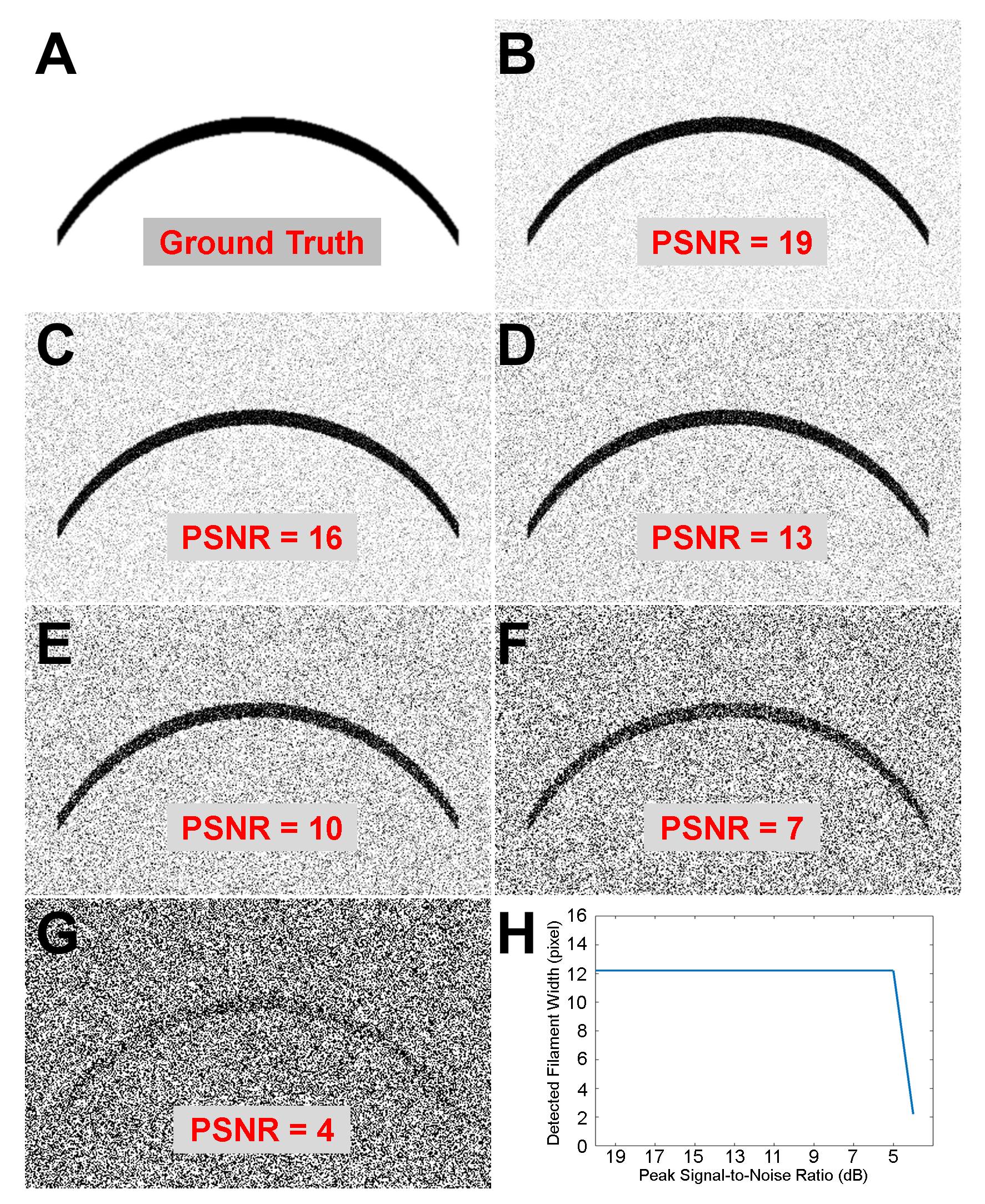

Supplement: Supplementary file 6 — Images for sensitivity analysis of filament width measurement. A) Synthetic ground truth image. B-G) Synthetic image with introduced noise at different levels. H) Detected filament length as a function of image noise (TIF 4838 kb). [file 12859_2017_1684_MOESM6_ESM.tif]

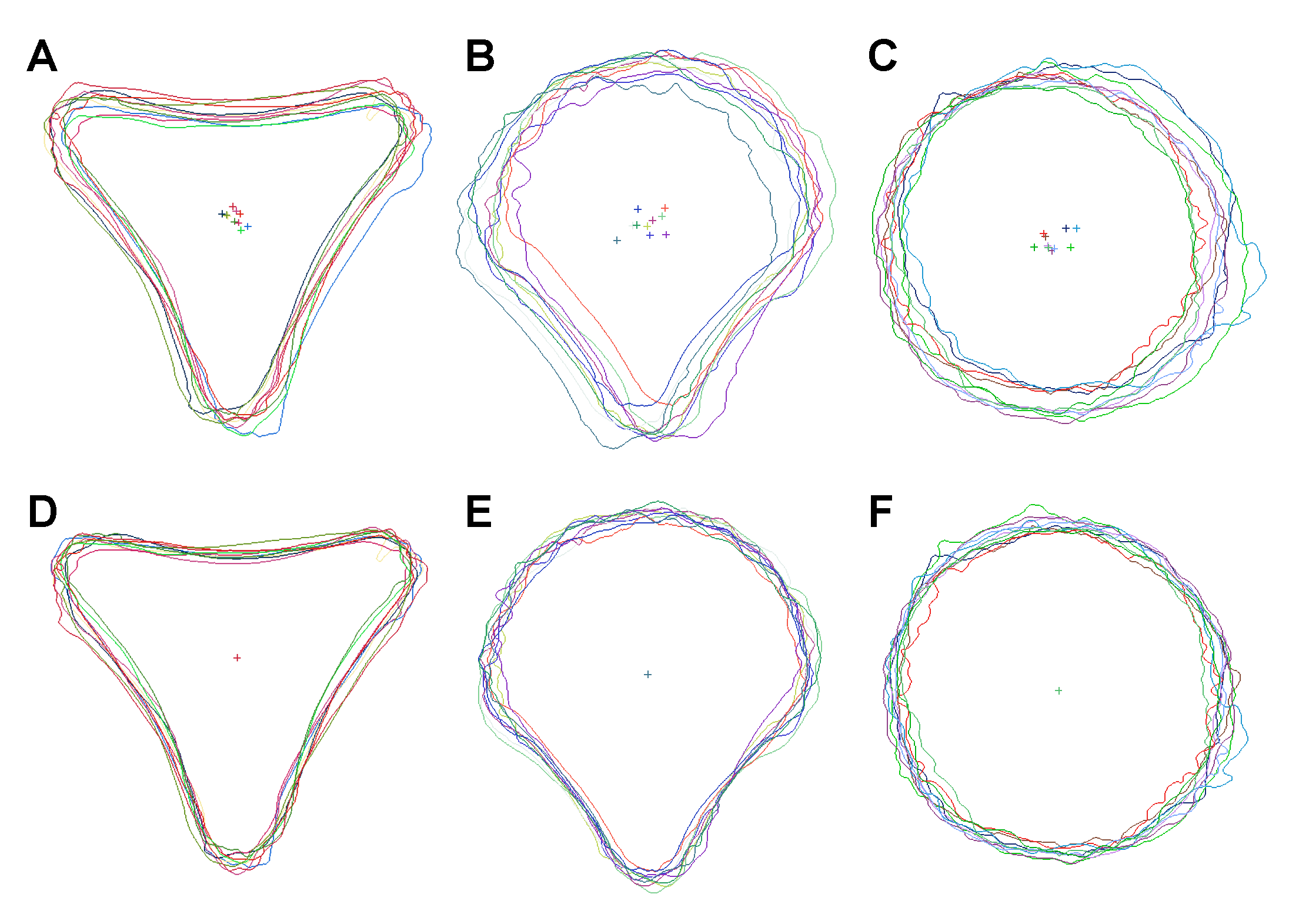

Supplement: Supplementary file 7 — Alignment of cells on micropatterns. A-C) Overlay of cell contours before alignment for Y, crossbow, and disc patterns, respectively. Crosses denote the center-of-mass (COM) of the cell regions. D-F) COM-based overlay of aligned cell contours. n = 10 for each micropattern (TIF 943 kb). [file 12859_2017_1684_MOESM7_ESM.tif]
