# Supplementary material for: An integrated enhancement and reconstruction strategy for the quantitative extraction of actin stress fibers from fluorescence micrographs
Source: BMC Bioinformatics. 2017 May 22;18:268. doi: 10.1186/s12859-017-1684-y (PMC5440974; doi:10.1186/s12859-017-1684-y)
Supplement: Supplementary file 2 — SupplementarySoftware_SFEX_BINF-D-16-00942. Stress Fiber Extractor (SFEX) 1.0. This file contains a test image and all the source code associated with the method proposed in this study (DOCX 6571 kb). [file 12859_2017_1684_MOESM2_ESM.docx]

**SFEX 1.0**

**User Manual**

**Content**

| 1. Preparation | 3 |
| --- | --- |
| 1.1 Source File | 3 |
| 1.2 Software Installation | 3 |
| 1.3 Data Directory | 3 |
| 2. Analysis of Thick Stress Fibers | 3 |
| 2.1 Image Enhancement and Segmentation | 3 |
| 2.1.1 Load Image | 3 |
| 2.1.2 Choose ROI | 4 |
| 2.1.3 Image Enhancement | 5 |
| 2.1.4 Binarization | 5 |
| 2.1.5 Remove Junction and Short Fragments | 6 |
| 2.1.6 Iterative Extraction of Linear Fragments | 6 |
| 2.1.7 Tip Registration | 7 |
| 2.2 Filament Reconstruction and Analysis | 7 |
| 2.2.1 Preview and Search Criteria | 7 |
| 2.2.2 Tip Pairing and Grouping | 7 |
| 2.2.3 Filament Sorting | 8 |
| 2.2.4 Filament Measurement | 8 |
| 3. Analysis of Stress Fibers Network | 8 |
| 3.1 Image Enhancement and Segmentation | 9 |
| 3.1.1 Load Image | 9 |
| 3.1.2 Choose ROI | 9 |
| 3.1.3 Image Enhancement | 10 |
| 3.1.4 Binarization | 10 |
| 3.1.5 Remove Junction and Short Fragments | 11 |
| 3.1.6 Iterative Extraction of Linear Fragments | 11 |
| 3.1.7 Tip Registration | 12 |
| 3.2 Filament Reconstruction and Analysis | 12 |
| 3.2.1 Preview and Search Criteria | 12 |
| 3.2.2 Tip Pairing and Grouping | 12 |
| 3.2.3 Filament Sorting | 13 |
| 3.2.4 Analysis | 13 |

**1. Preparation**

**1.1 Source File**

Download the software package named SFEX.zip and unzip it (Fig. 1). The unzipped folder contains the following files. The testing image is named *Crossbow.tiff*.


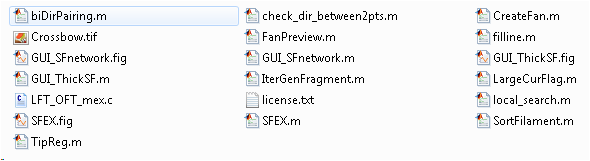


Figure 1. Files included with SFEX 1.0 package

**1.2 Software Installation**

In SFEX, the majority of the codes were written using Matlab except for the routine for image enhancement which was written in *C*, *LFT_OFT_mex.c*. In order to call this external *C* function, users need to create a MEX file by setting up a *C* compiler to compile the *C* code via the command '*mex -setup*'. A list of supported and compatible compilers can be found in the following link.

<https://www.mathworks.com/support/sysreq/previous_releases.html>

Upon successful compilation, users should be able to see a new file (e.g. *LFT_OFT_mex.mexw64*) created in the folder.

**1.3 Data Directory**

While using this software, two new folders (*data* and *result*) will be created containing all necessary parameters, intermediate and ultimate results.

(1) *data*. This folder contains all intermediate and ultimate results in *.mat* format.

(2) *result*. All exported data sheets (in *.xlsx* format) are saved in this folder.

**2. Analysis of Thick Stress Fibers**

**2.1 Image Enhancement and Segmentation**

***2.1.1 Load Image***

To start, run the script, *SFEX.m* to load the first GUI (Fig. 2) and click [1] to launch a new GUI for analyzing thick stress fibers (Fig. 3).

Click [1] (Fig. 3) to go back to the main GUI.

Click [2] (Fig. 3) to load the image.


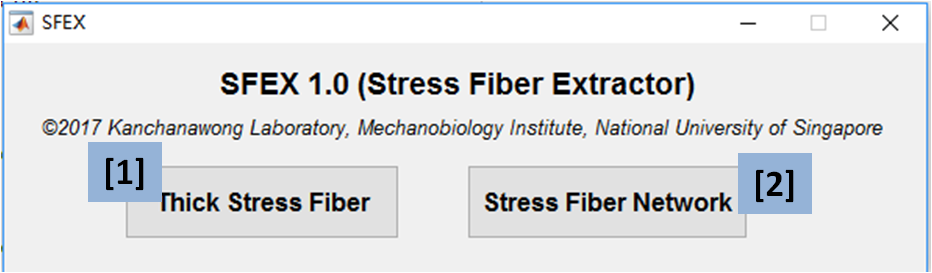


Figure 2. First GUI of SFEX


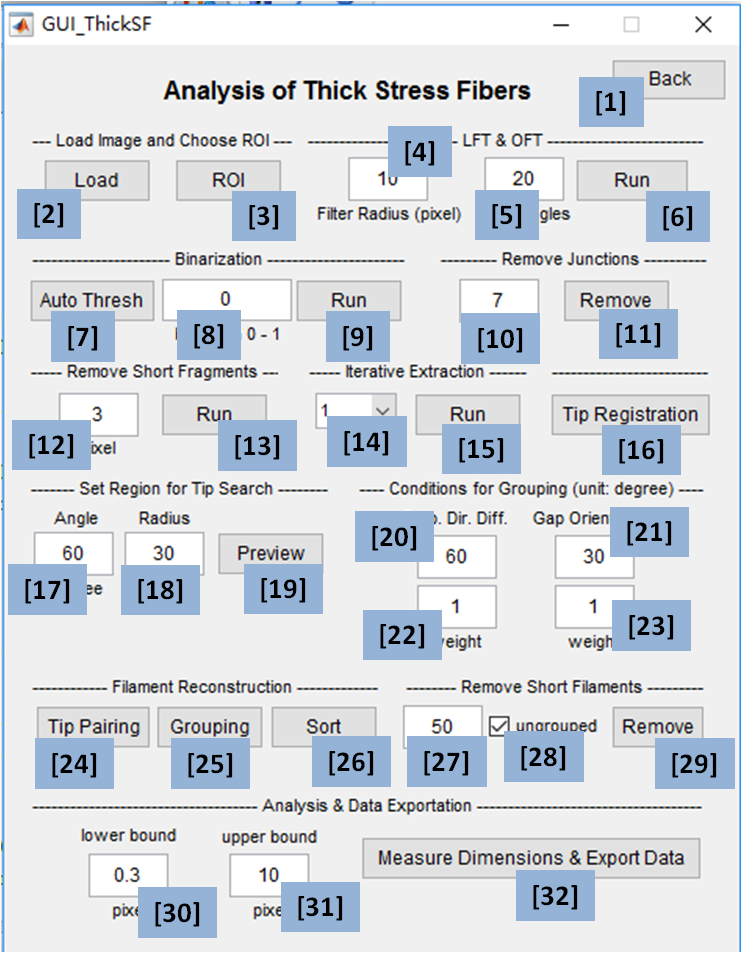


Figure 3. GUI for analysis of thick stress fibers

***2.1.2 Choose ROI***

Click [3] (Fig. 3) choose the region of interest (ROI). To do this, left-single-click all neighboring control points. Right-single-click at the last control point, the region will close itself (Fig. 4, left). Left-click the center of the ROI twice to save the ROI.


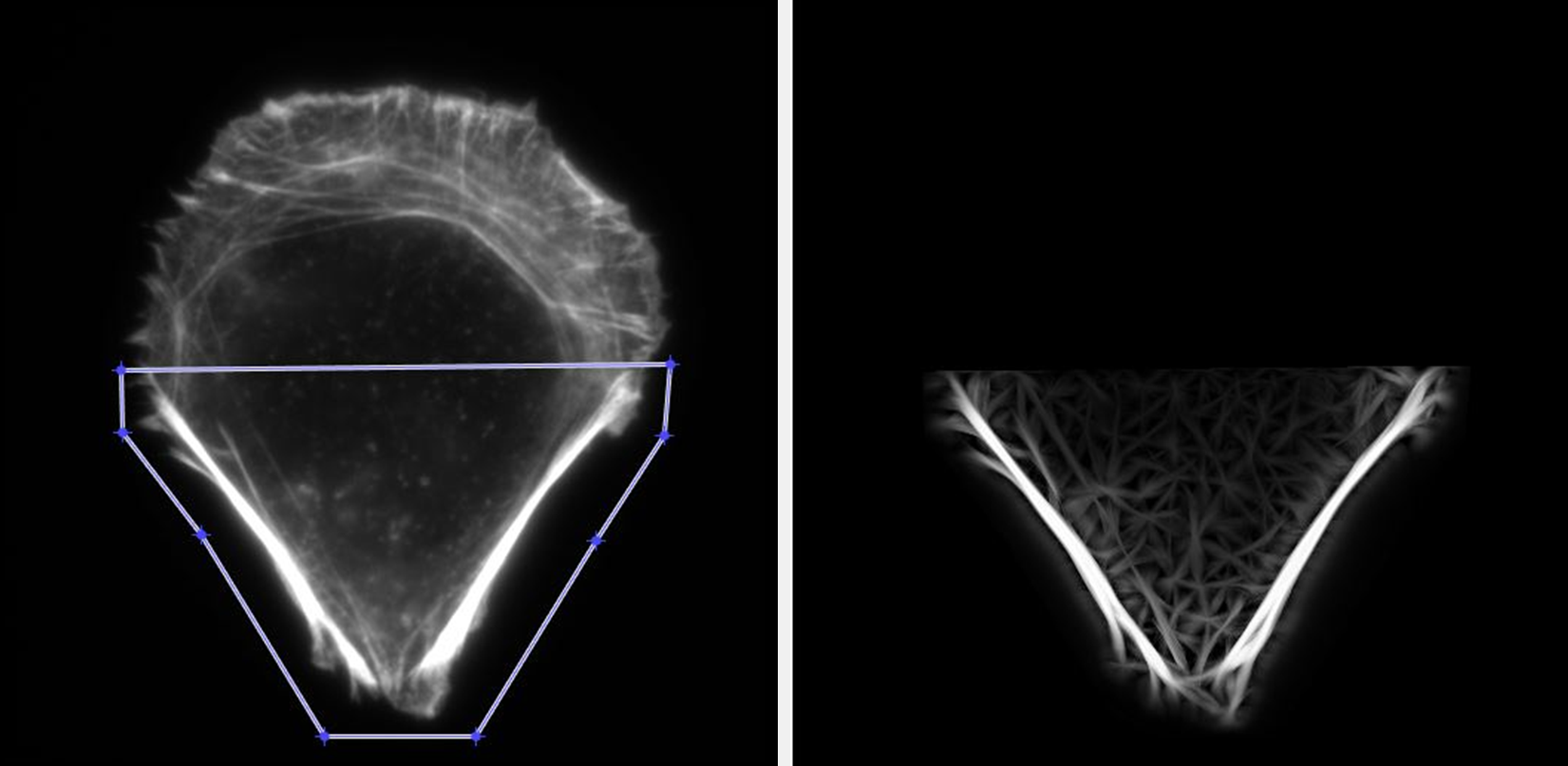


Figure 4. Analysis of thick stress fibers: Enhanced thick stress fibers in ROI

***2.1.3 Image Enhancement***

The image enhancement method we use in our algorithm is line and orientation filter transform (LFT and OFT). For this enhancement approach, users should define the radius and number of rotations of the scanning line segment at [4] and [5] (Fig. 3).

Click [6] to run LFT and OFT, and visualize the enhanced image (Fig. 4, right).

***2.1.4 Binarization***

Click [7] (Fig. 3) to automatically calculate the threshold using Otsu's method for binarizing the enhanced image whose intensities have been normalized to 1 and the threshold will appear at [8] (Fig. 3). The binary image (Fig. 5, left) and overlay of original image and extracted skeleton (Fig. 5, right) will appear for users to evaluate by observation. Users can manually define the threshold value between 0 and 1 at [8] (Fig. 3) as needed and click [9] (Fig. 3) to assess again.

** Users can zoom in to see details of figures.*

** Users are not required to use Otsu's threshold. This serves primarily to provides us an initial value to start with.*


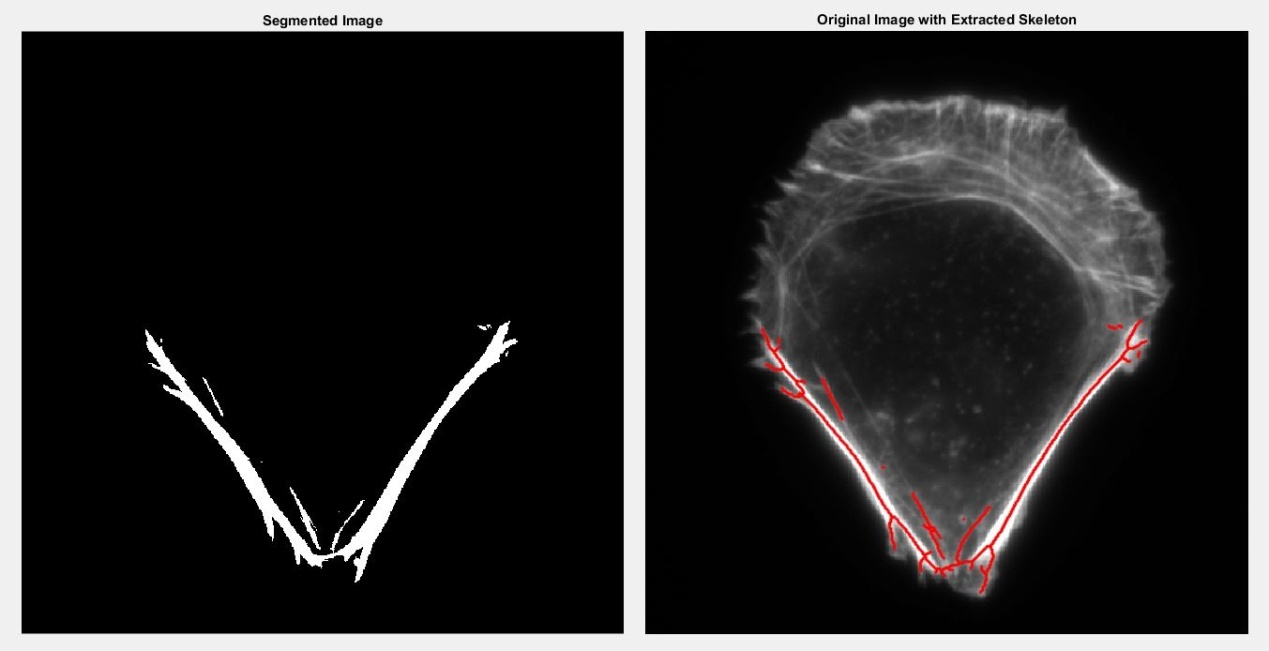


Figure 5. Analysis of thick stress fibers: Skeleton of binarized image.

***2.1.5 Remove Junction and Short Fragments***

To create a pool of minimal linear filament fragments, regions of junctions should be removed. Click [11] (Fig. 3) to remove a local region of *n*-by-*n* pixels around each junction (Fig. 6, left). This step will also remove single points. It is suggested to remove some short filament fragments primarily generated from noise by clicking button [13] (Fig. 3) (Fig. 6, right).

** Users may feel free to define the size of the junction region at [10] (Fig. 3) and minimal number of pixels in each filament fragment at [12] (Fig. 3).*


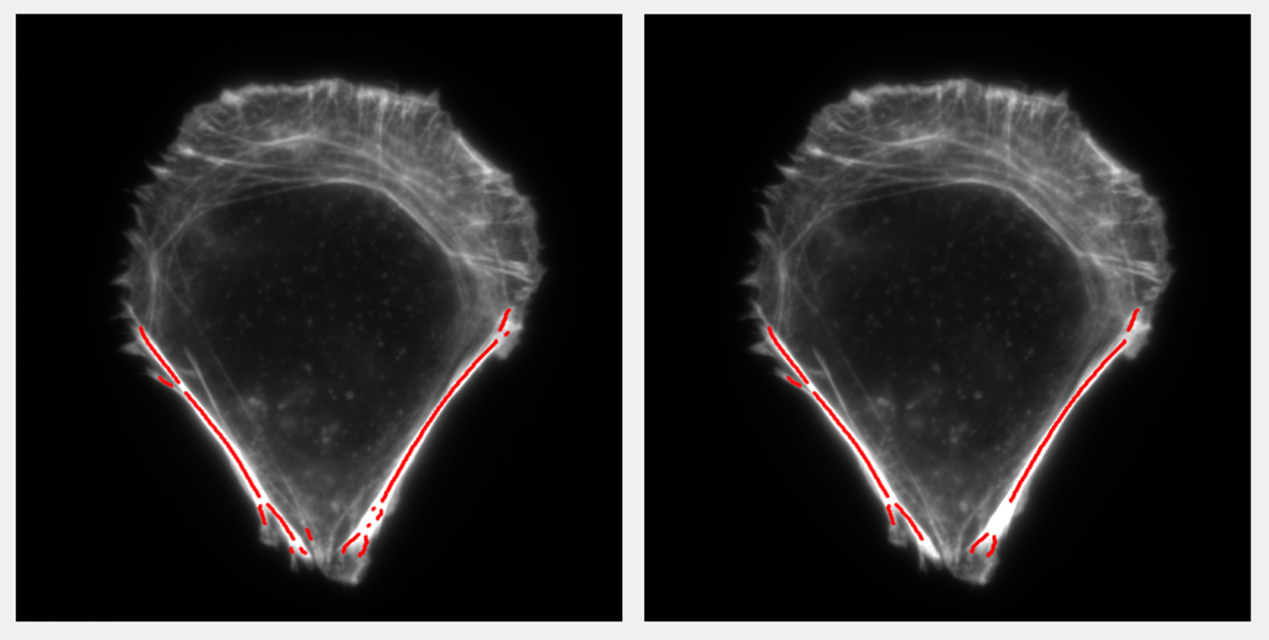


Figure 6. Analysis of thick stress fibers: Skeleton with junction and short fragments removed

***2.1.6 Iterative Extraction of Linear Fragments (Optional)***

Although this step is optional our result has shown that an iterative extraction of filament fragments will significantly recover undetected linear structures, especially in highly complex filament networks. Choose the number of additional iterations (from 1 to 5) you want to perform at [14] (Fig. 3) and click [15] (Fig. 3). If you choose to iteratively extract filament fragments, the command window will display its progress including the iteration you are doing and number of fragments added.

** For analysis of thick stress fibers, this step can be skipped.*

***2.1.7 Tip Registration***

To register the propagation direction of each tip, users can click [16] (Fig. 3). Propagation directions of some fragment endpoints will be shown (Fig. 7, left).


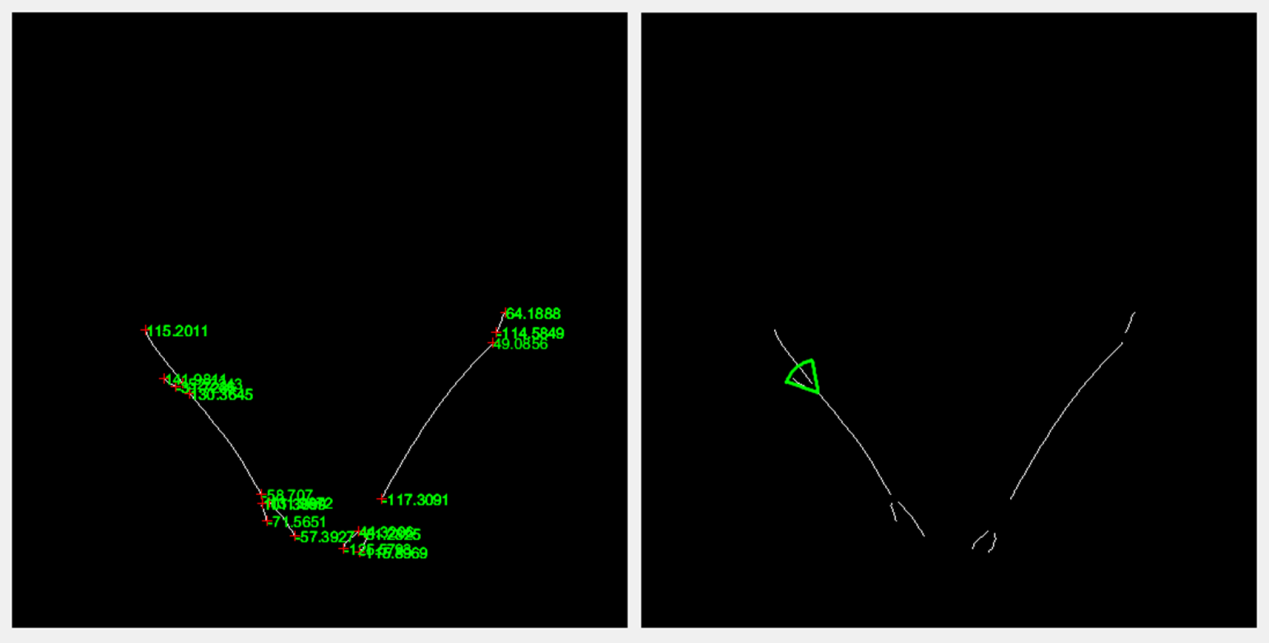


Figure. 7 Analysis of thick stress fibers: Display of propagation directions and preview of search area

**2.2 Filament Reconstruction and Analysis**

***2.2.1 Preview and Search Criteria***

Enter the values for the search angle and radius at [17] and [18] (Fig. 3). Click [19] (Fig. 3) to preview the search region and check whether it is suitable to cover most gaps that should be filled. An image of filament fragments will appear requesting users to click one location of the network for preview (Fig. 7, right).

Enter the maximum allowable orientation difference between two endpoints at [20] (Fig. 3) and the maximum allowable angle difference between base endpoint and gap vector at [21] (Fig. 3).

Enter the weights for similarity and continuity conditions during scoring calculation at [22] and [23] (Fig. 3).

***2.2.2 Tip Pairing and Grouping***

Click [24] (Fig. 3) to pair endpoints.

Click [25] (Fig. 3) to generate composite filaments.

***2.2.3 Filament Sorting***

Click [26] (Fig. 3) to sort reconstructed filaments

You may define the minimum filament length allowed at [27] (Fig. 3) and toggle at [28] (Fig. 3) to choose whether you want to remove ungrouped filament fragments.

** Tip: To remove ungrouped fragments.*

Click [29] to perform the above step and visualize the overlay of original image and centerlines of detected filaments shown in randomly generated colors (Fig. 8, left).


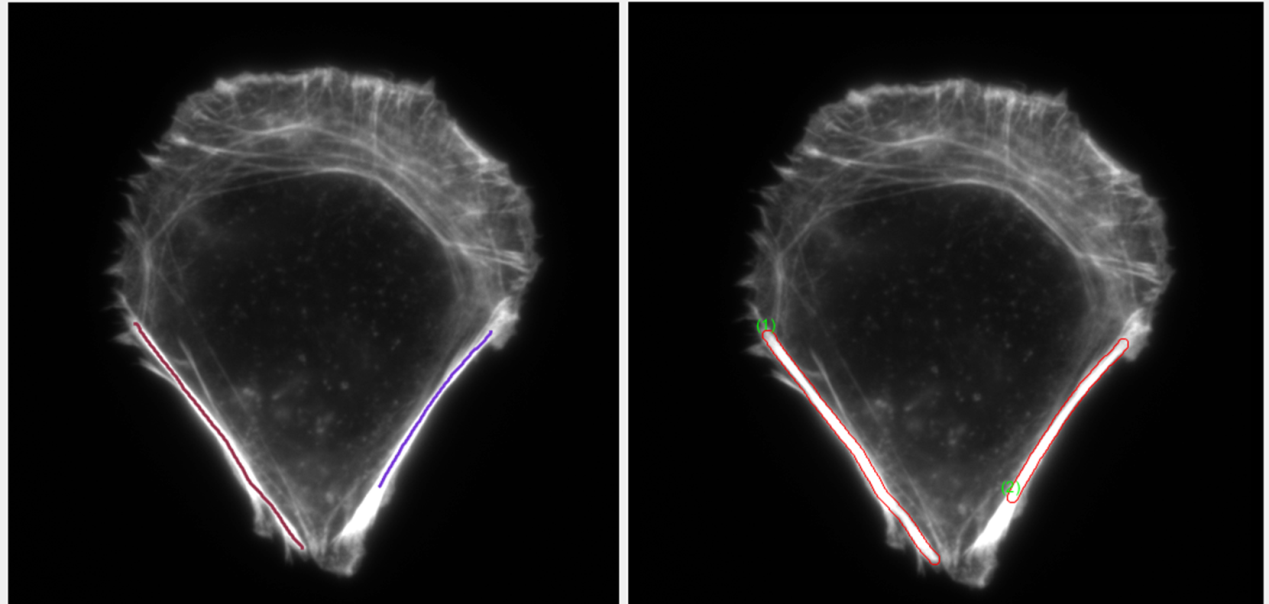


Figure 8. Analysis of thick stress fibers: Contours of detected thick stress fibers

***2.2.4 Filament Measurement***

Before measuring the dimensions of detected filaments by clicking [32] (Fig. 3), users must define distance map-based bounds at [30] and [31] (Fig. 3) for measuring their average width. The filament contours with their determined width will be shown (Fig. 8, right).

This step also generates filament information into excel sheets (*ThickSF_Info.xlsx* in *result* folder) as shown in Fig. 9.


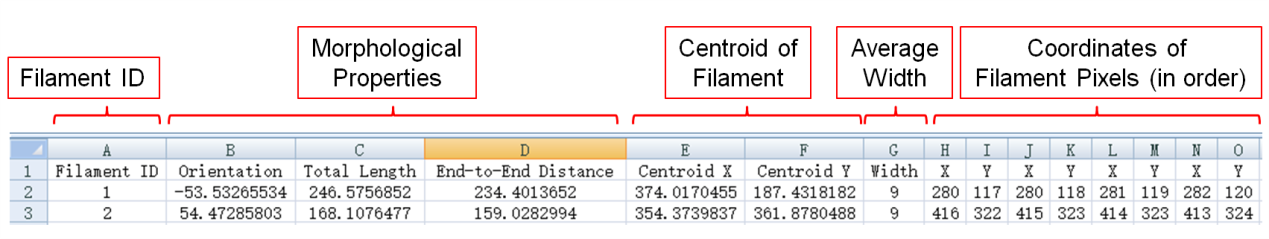


Figure 9. Analysis of thick stress fibers: Exported excel sheet of filament information

**3. Analysis of Stress Fibers Network**

Click [2] in Figure. 2 to launch a new GUI for analyzing stress fiber network (Fig. 10).


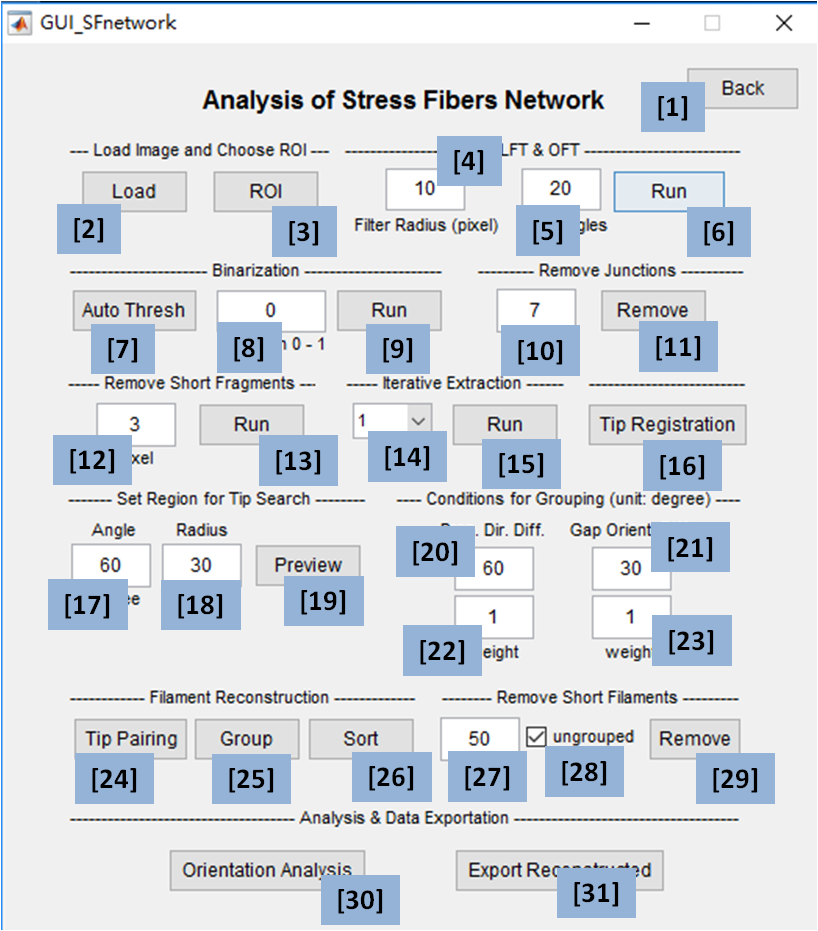


Figure 10. GUI for analysis of stress fibers network

**3.1 Image Enhancement and Segmentation**

***3.1.1 Load Image***

Click [1] (Fig. 10) to go back to the main GUI.

Click [2] (Fig. 10) to load the image.

***3.1.2 Choose ROI***

Click [3] (Fig. 10) to choose the region of interest (ROI). To do this, left-single-click all neighboring control points. Right-single-click at the last control point, the region will close itself (Fig. 11, left). Left-click the center of the ROI twice to save the ROI.


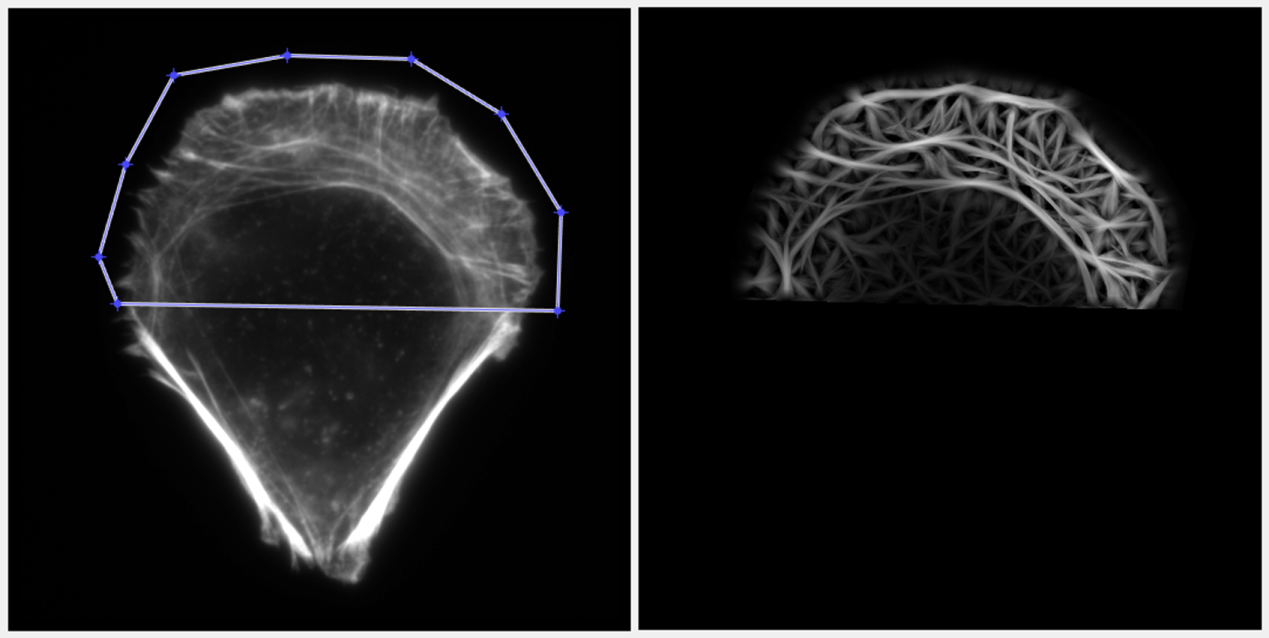


Figure 11. Analysis of stress fibers network: Enhanced stress fiber network in ROI

***3.1.3 Image Enhancement***

The image enhancement method we used in our algorithm is line and orientation filter transform (LFT and OFT). For this enhancement approach, users should define the radius and number of rotations of the scanning line segment at [4] and [5] (Fig. 10).

Click [6] to run LFT and OFT and visualize the enhanced image (Fig. 11, right).

***3.1.4 Binarization***

Click [7] (Fig. 10) to automatically calculate the threshold using Otsu's method for binarizing the enhanced image whose intensities have been normalized to 1 and the threshold will appear at [8] (Fig. 10). The binary image (Fig. 12, left) and overlay of original image and its extracted skeleton (Fig. 12, right) will appear for users to evaluate by observation. Users may feel free to manually define the threshold value between 0 and 1 at [8] (Fig. 10) and click [9] (Fig. 10) to assess again.

** Users may feel free to zoom in to see details of figures.*

** Users are not required to use Otsu's threshold, as this serves primarily as an initial value to start with.*


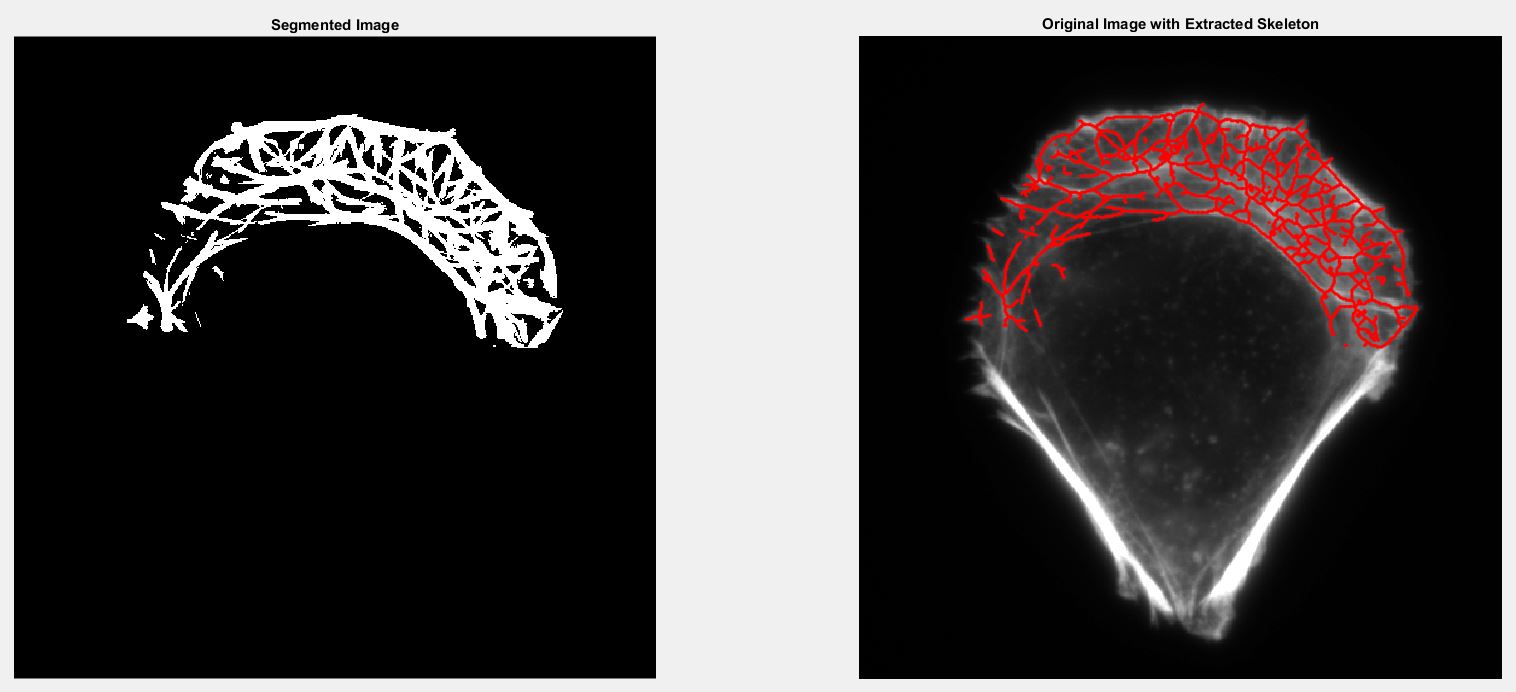


Figure 12. Analysis of stress fibers network: Skeleton of binarized image

***3.1.5 Remove Junction and Short Fragments***

To create a pool of minimal linear filament fragments, regions of junctions should be removed. Click [11] (Fig. 10) to remove a local region of *n*-by-*n* pixels around each junction (Fig. 13, left). This step will also remove single points. It is suggested to remove some short filament fragments primarily generated from noise. This can be done by clicking [13] (Fig. 10) (Fig. 13, right).

** Users may feel free to define the size of the junction region at [10] (Fig. 10) and minimal number of pixels in each filament fragment at [12] (Fig. 10).*


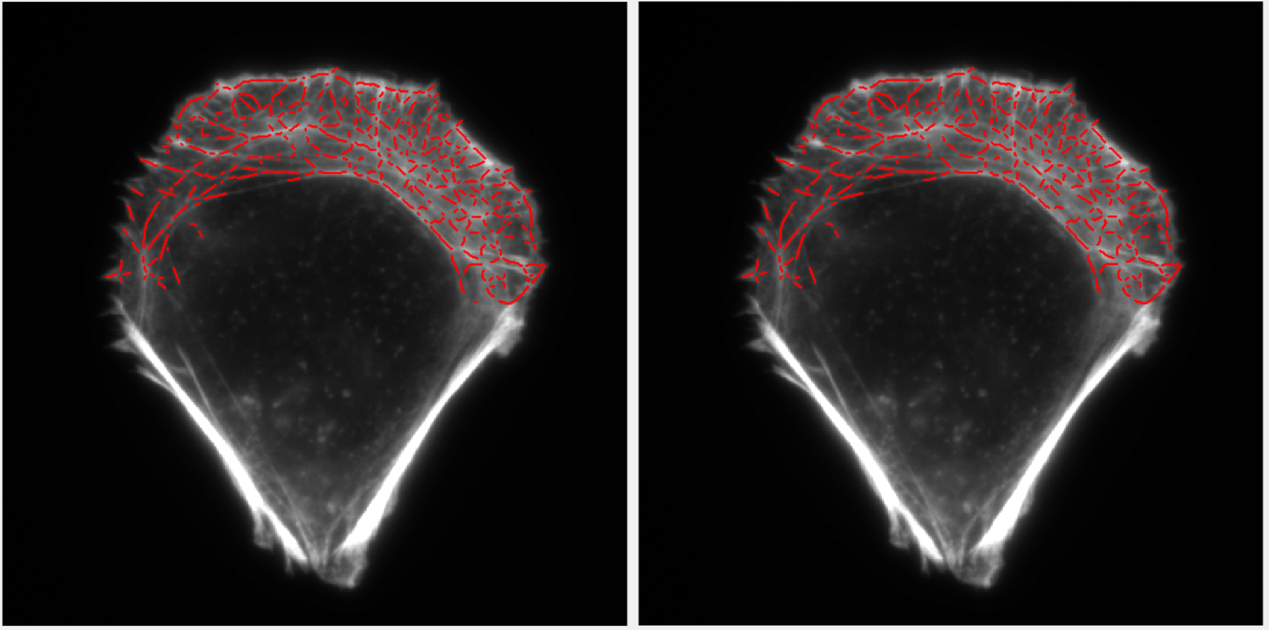


Figure 13. Analysis of stress fibers network: Skeleton with junction and short fragments removed

***3.1.6 Iterative Extraction of Linear Fragments (Optional)***

Although this step is optional, our result has shown that an iterative extraction of filament fragments will significantly recover undetected linear structures, especially in highly complex filament networks. Choose the number of additional iterations (from 1 to 5) you want to perform at [14] (Fig. 10) and click [15] (Fig. 10). If you choose to iteratively extract filament fragments, the command window will display its progress including the iteration you are doing and number of fragments added.

***3.1.7 Tip Registration***

To register the propagation direction of each tip, users can click [16] (Fig. 10). Propagation directions of some fragment endpoints will be shown (Fig. 14, left).


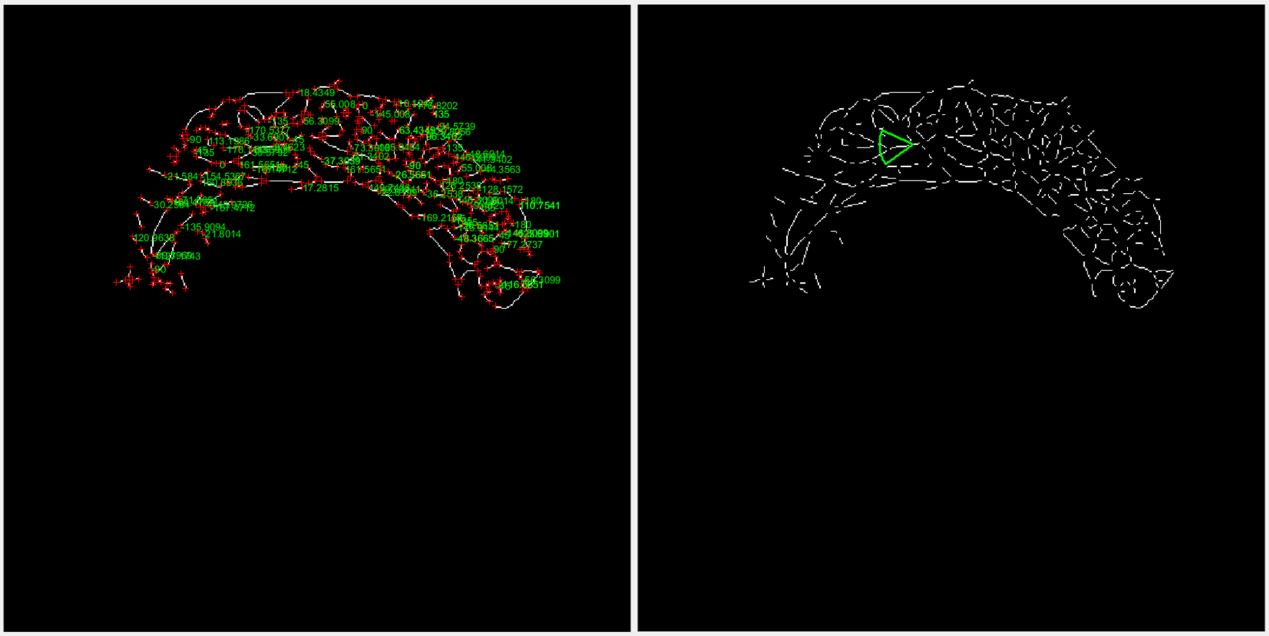


Figure 14. Analysis of stress fibers network: Display of propagation directions and preview of search area

**3.2 Filament Reconstruction and Analysis**

***3.2.1 Preview and Search Criteria***

Enter the values for the search angle and radius at [17] and [18] (Fig. 10).

Click [19] (Fig. 10) to preview the search area and check whether it is suitable to cover most gaps that should be filled. An image of filament fragments will appear requesting users to click one location of network for preview (Fig. 14, right).

Enter the values for the maximum allowable orientation difference between two endpoints at [20] (Fig. 10) and the maximum allowable angle difference between base endpoint and gap vector at [21] (Fig. 10).

Enter the weights for similarity and continuity conditions during scoring calculation at [22] and [23] (Fig. 10).

***3.2.2 Tip Pairing and Grouping***

Click [24] (Fig. 10) to pair endpoints.

Click [25] (Fig. 10) to generate composite filaments.

***3.2.3 Filament Sorting***

Click [26] (Fig. 10) to sort reconstructed filaments

You can define the minimum filament length allowed at [27] (Fig. 10) and toggle at [28] (Fig. 10) to choose whether you want to remove ungrouped filament fragments.

** Tip: To remove ungrouped fragments.*

Click [29] to perform the above step and visualize the overlay of original image and centerlines of detected filaments shown in randomly generated colors (Fig. 15).


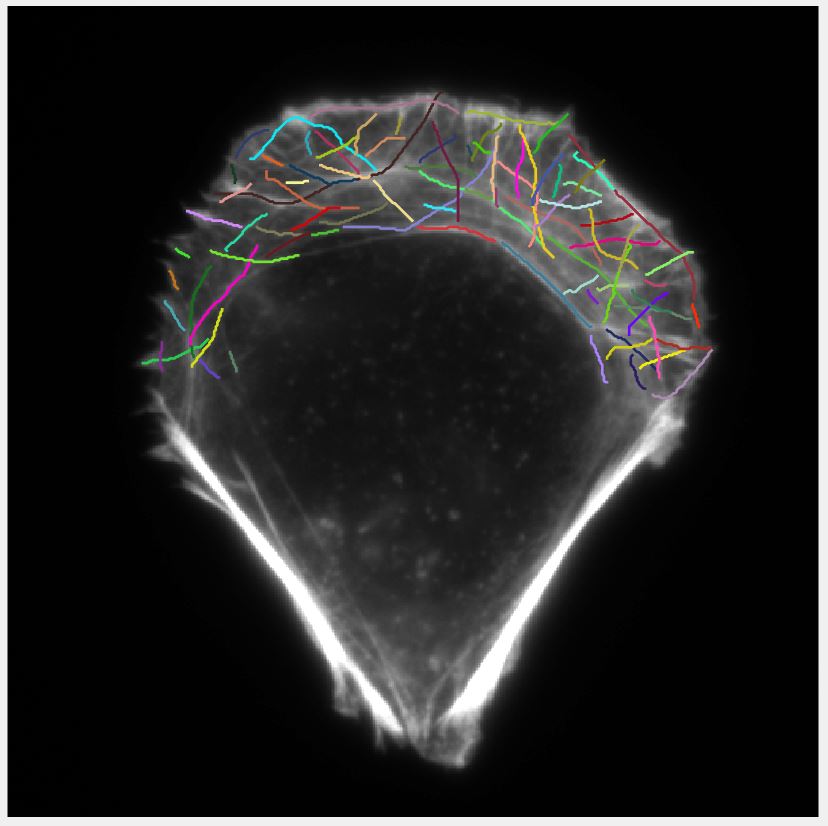


Figure 15. Analysis of stress fibers network: Detected stress fiber network

***3.2.4 Analysis***

Users may check the distribution of F-actin orientations by clicking [30] (Fig. 10) to export relevant information into excel sheet (*SFnetwork_OrientationInfo.xlsx* in *result* folder) as shown in Figure 16.

** Noted that this analysis is performed using all minimal linear fragments with junctions removed.*


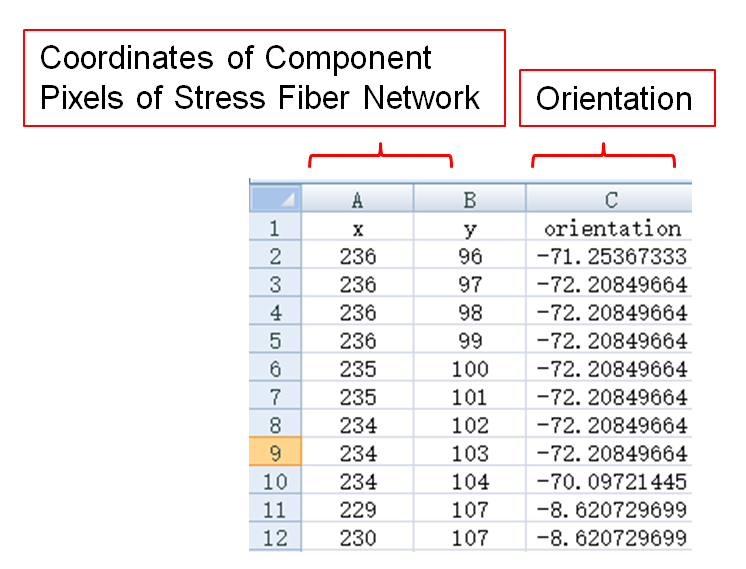


Figure 16. Analysis of stress fibers network: Exported information of F-actin orientation

Click [31] (Fig. 10) to generate filament information into excel sheets (*SFnetwork_Info.xlsx* in *result* folder) as shown in Fig. 17.


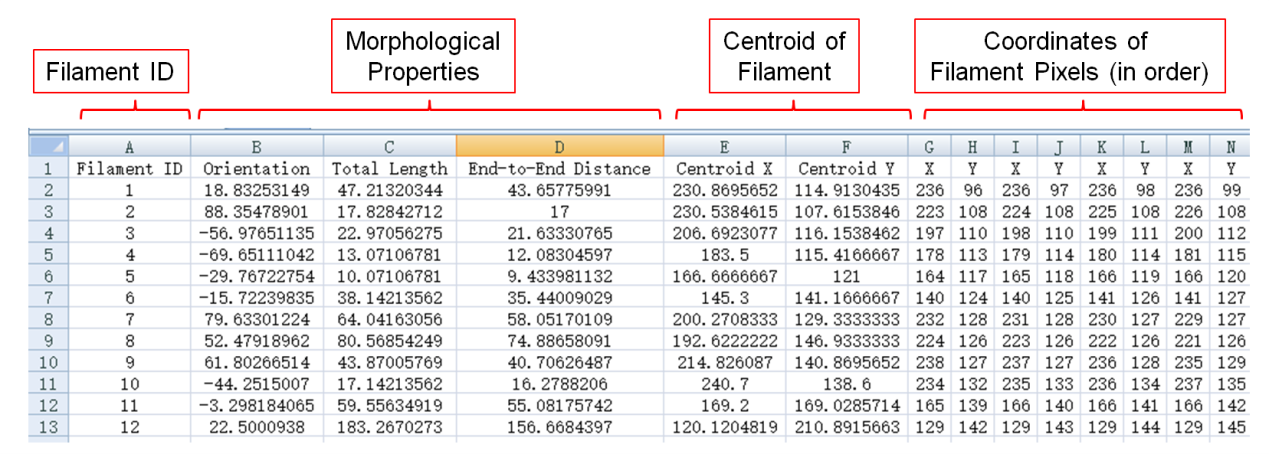


Figure 17. Analysis of stress fibers network: Exported information of reconstructed filaments

Contact Information and Updates

Contact 1: Dr Pakorn Kanchanawong (biekp@nus.edu.sg)

Contact 2: Zhen Zhang (a0045820@u.nus.edu)

<https://www.nanoscalemechanobiology.org/>

<https://github.com/KanchanawongLab>
